# Improved Morphological and Localized Surface Plasmon Resonance (LSPR) Properties of Fully Alloyed Bimetallic AgPt and Monometallic Pt NPs Via the One-Step Solid-State Dewetting (SSD) of the Ag/Pt Bilayers

**DOI:** 10.1186/s11671-019-3170-0

**Published:** 2019-10-24

**Authors:** Sundar Kunwar, Puran Pandey, Sanchaya Pandit, Mao Sui, Jihoon Lee

**Affiliations:** 1 Department of Electronic Engineering, College of Electronics and Information, Kwangwoon University, Nowon-gu, Seoul 01897 South Korea; 2 Institute of Hybrid Materials, College of Materials Science and Engineering, Qingdao University, Qingdao, 266071 People’s Republic of China

**Keywords:** Plasmonics, Monometallic, Bimetallic, Nanoparticles, Solid-state dewetting, Ag sublimation

## Abstract

Multi-metallic alloy nanoparticles (NPs) can offer a promising route for the integration of multi-functional elements by the adaptation of advantageous individual NP properties and thus can exhibit the multi-functional dynamic properties arisen from the electronic heterogeneity as well as configurational diversity. The integration of Pt-based metallic alloy NPs are imperative in the catalytic, sensing, and energy applications; however, it usually suffers from the difficulty in the fabrication of morphologically well-structured and elementally well-alloyed NPs, which yields poor plasmonic responses. In this work, the improved morphological and localized surface plasmon resonance (LSPR) properties of fully alloyed bimetallic AgPt and monometallic Pt NPs are demonstrated on sapphire (0001) via the one-step solid-state dewetting (SSD) of the Ag/Pt bilayers. In a sharp contrast to the previous studies of pure Pt NPs, the surface morphology of the resulting AgPt and Pt NPs in this work are significantly improved such that they possess larger size, increased interparticle gaps, and improved uniformity. The intermixing of Ag and Pt atoms, AgPt alloy formation, and concurrent sublimation of Ag atoms plays the major roles in the fabrication of bimetallic AgPt and monometallic Pt NPs along with the enhanced global diffusion and energy minimization of NP system. The fabricated AgPt and Pt NPs show much-enhanced LSPR responses as compared to the pure Pt NPs in the previous studies, and the excitation of dipolar, quadrupolar, multipolar and higher-order resonance modes is realized depending upon the size, configuration, and elemental compositions. The LSPR peaks demonstrate drastic alteration along with the evolution of AgPt and Pt NPs, i.e., the resonance peaks are shifted and enhanced by the variation of size and Ag content.

## Background

In the last decade, metallic nanoparticles (NPs) have received a great deal of research attentions due to their various applicability in electronics, photonics, energy, catalysis, sensors, storages, and biomedicines [1–7]. Especially, the alloy NPs with the multiple metallic elements are of an interesting class of materials because of their multi-functionality and dynamic properties arisen from the heterogeneous properties as well as geometrical diversities [8–10]. As just one example, the hydrogen peroxide (H_2_O_2_) sensitivity has been significantly improved by the incorporation of AuAg alloy NPs on Cu_2_O nanocubes as compared to the Cu_2_O, Ag–Cu_2_O, and Au–Cu_2_O, which can be attributed to the high electrical conductivity and chemical stability of the Au and Ag alloy in the NP dimension [11].

The capability of metallic NPs to interact with the photons of various wavelengths induces the localized surface plasmon resonance (LSPR), which has tremendously scaled up the range of functionality and applicability of NPs [12–14]. The LSPR is a collective oscillation of electrons on the plasmonic NP surface in resonance with the incident photons, which results in the strong absorption or scattering of light as well as intensification of the electromagnetic fields around the NPs. As the LSPR effect of metallic NPs is closely related to the size, shape, composition, and metal-support interactions, it can be readily exploited to enhance/alter the electrical, magnetic, optical and catalytic properties for the corresponding devices [15]. The LSPR property of metallic NPs has been extensively investigated for enhancing the light absorption in thin film solar cells, photodetectors, and stealth technology [16, 17]. Meanwhile, achieving LSPR from the metallic NPs in the broadband wavelength is still challenging as well as demanding in various applications such as perfect absorbers in solar cell applications [18, 19].

Particularly, the Ag NPs have been widely utilized for various plasmonic applications owing to their strong LSPR properties and electrical conductivity [16, 20] and at the same time, the Pt NPs have been extensively employed in the catalytic applications due to their strong catalytic activity, selectivity, and corrosion resistance [21]. In this regard, the AgPt alloy NPs can be promising candidates for the plasmon-based catalysts, fuel cells, chemo/biosensors and photonic applications, which can successfully integrate the advantages of Ag and Pt with the added design flexibility. In addition, the absorption bandwidth can be enlarged as the LSPR occur at a different wavelength for Ag and Pt such as Ag is mostly active in the VIS while Pt is in the UV region. To exploit the applications of AgPt alloy NPs, it is necessary to understand the growth, structural and optical behaviors of the alloy NPs in detail, which however has not been explored in detail yet.

In this work, the fabrication of bimetallic AgPt and monometallic Pt NPs is demonstrated by the solid-state dewetting (SSD) on sapphire (0001). The SSD is a simple yet very clever approach for the fabrication of substrate supported metallic nanostructures [22–24]. The introduction of Ag component into the Pt matrix can achieve the dual advantages of enhanced dewetting process and improved LSPR properties due to the high diffusivity of Ag atoms and strong interaction with the photons simultaneously. Based on the enhanced dewetting of Ag/Pt bilayer and sublimation of Ag atoms at high temperature, a variety of well-structured, isolated, and uniform AgPt and Pt NPs are demonstrated by controlling the initial bilayer compositions and growth parameters. Depending upon the size, uniformity, and elemental composition, these AgPt and Pt NPs exhibit significantly improved and dynamic LSPR responses in the UV-VIS-NIR wavelength as compared to the pure Pt NPs in the previous studies.

## Methods

### Sample and Bilayer Preparations

In this work, the double-sided polished 430-μm-thick sapphire (0001) wafers with ± 0.1° off axis were used as substrates. First, the 6 × 6 mm^2^ diced sapphire sample was degassed at 600 °C for 30 min under 1 × 10^−4^ Torr to ensure the removal of surface contaminants, particulates, and trapped gases. The surface morphology, reflectance, and transmittance spectra of bare sapphire are shown in Additional file 1: Figure S1 and the surface texture of sapphire was smooth with the average height below the sub nanometer scale after the degassing. In the next step, the Ag and Pt films were sequentially deposited on a cleaned sapphire using the respective metallic targets of 99.999% purity as shown in Additional file 1: Figure S1(c). The Ag layer was first deposited on sapphire and then the Pt layer deposited on top, named as Ag _(*a* nm)_/Pt _(*b* nm)_ bilayer having (*a* + *b*) nm total thickness. Reversed case with the Pt layer first can result in reduced intermixing of atoms due to the lower diffusivity of Pt atoms. To analyze the effect of bilayer thickness on the growth of nanostructures, the total thickness as well as individual layer thickness were systematically varied and three series of samples were fabricated in total, namely, Ag_7nm_/Pt_1.5nm_, Ag_5nm_/Pt_2.5nm_, and Ag_21nm_/Pt_4.5nm_ bilayers. The deposition rate was maintained at 0.05 nm/s for both metallic films by a plasma-assisted sputtering with 5 mA ionization current under 1 × 10^−1^ Torr and thickness was controlled by the duration of deposition (e.g., 20 s equal to 1 nm).

### Nanostructure Fabrication

To fabricate various AgPt and Pt nanostructures, each sample in an Ag/Pt bilayer series was systematically annealed at temperatures between 500 °C and 900 °C with the 50 °C interval. Prior to the annealing, the Inconel blank was placed on the backside of the sample to ensure the radiative thermal conduction and the annealing process was performed in a pulse laser deposited (PLD) chamber under 1 × 10^−4^ Torr. The designated temperatures were reached by the ramping rate at 4 °C/s and then kept constant for 120 s for all samples to ensure the matured Ostwald ripening of nanostructures. In order to maintain consistency, the overall annealing process was controlled by a computer program and to terminate the growth, the heating system was turned off while keeping the chamber vacuum until the temperature was reduced to an ambient over time.

### Characterization and Simulation

The surface morphology of fabricated AgPt and Pt nanostructures was investigated using a non-contact mode of atomic force microscope (NC-AFM, Park Systems, South Korea) and the XEI software was utilized for the detailed analysis of size, shape, and density of NPs. Scanning electron microscope (SEM, COXEM, South Korea) was employed to investigate the AgPt and Pt nanostructures on a large scale and the elemental analysis were performed by using an energy-dispersive X-ray spectroscope (EDS, Thermo Fisher, Noran System 7, USA). The optical spectra of AgPt and Pt nanostructures were acquired by using a NOST system (Nostoptiks, South Korea), equipped with the ANDOR sr-500i spectrograph, CCD detector, optical microscope, and with the excitation from the halogen and deuterium lamps. The finite difference time domain (FDTD, Lumerical Solutions, Canada) was employed to simulate the e-field distributions and extinction of NPs, and the refractive index of sapphire, Ag, and Pt were fitted to the Rakic and Palik models within the spectral range of 250–1100 nm [25, 26]. For the binary alloy compositions, the dielectric constants of pure Ag and Pt were averaged based on the atomic percentage (at %) fraction [27]. For example, the dielectric constant of Ag–Pt varies along with the averaged value of at. % fraction based on the ellipsometry measurement. Thus, the dielectric constant of Au-Pt was constructed by the averaging of the at. % fraction. The detailed simulation setup is presented in Additional file 1: Figure S1(d).

## Results and Discussion

**Fig. 1:**
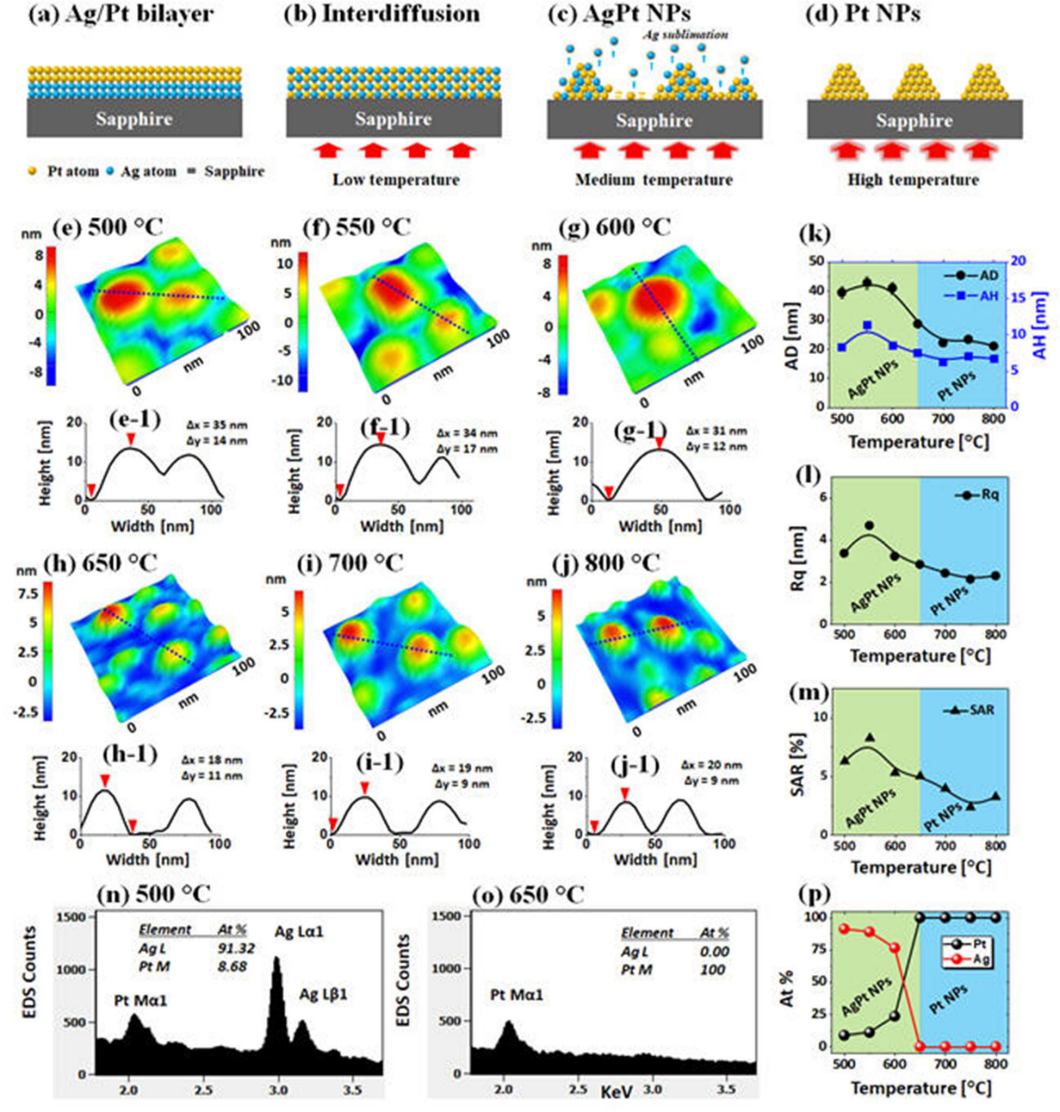
Fabrication of AgPt and Pt NPs with the Ag_7nm_/Pt_1.5nm_ bilayers by annealing between 500 and 800 °C for 120 s. (**a**) – (**d**) Schematic illustration of the formation of AgPt and Pt NPs from the Ag/Pt bilayers. (**e**) – (**j**) Atomic force microscope (AFM) side-views. (**e**-**1**) – (**j**-**1**) Cross-sectional line profiles across the typical NPs in (**e**) – (**j**). (**k**) Summary plots of average diameter (AD), average height (AH). (**l**) – (**m**) RMS roughness (Rq) and surface area ratio (SAR). (**n**)-(**o**) Energy dispersive x-ray spectroscopy (EDS) spectra of the samples annealed at 500 and 650 °C. (**p**) Summary plots of atomic (at) % of Pt and Ag

Figure 1 shows the evolution of AgPt and Pt NPs fabricated from the Ag_7nm_/Pt_1.5nm_ bilayer series as a function of annealing temperature between 500 and 800 °C for 120 s. The systematic annealing of the Ag_7nm_/Pt_1.5nm_ bilayer series demonstrated well-developed AgPt and Pt nanostructures based on the solid-state dewetting (SSD) and the gradual evolution of size, spacing, areal density, and elemental variation of isolated semi-spherical NPs were observed at various temperatures. The growth of isolated NPs from the continuous bilayer can be explained based on the thermal energy-induced SSD mechanism [28, 29]. The dewetting kinetics and thus structure, configurations, and arrangement of NPs can be directly determined by the control of growth parameters such as annealing temperature, initial film thickness, and substrate properties [30, 31]. In the SSD approach, the deposited films are adequately heated under controlled conditions to achieve various sizes and configuration of NPs based on the surface diffusion, interdiffusion, energy minimization, and equilibrium configurations. Meanwhile, the bilayer dewetting process can be further complicated due to the differences in the diffusivity of atoms, surface energies, interdiffusion, miscibility, and interaction with substrate [32, 33].

In this study, the Ag was chosen as an underlayer because of its high surface diffusivity and low-surface energy as compared to the Pt atoms [34], which can facilitate the overall dewetting process of Ag/Pt bilayer and thus can form well-structured and isolated AgPt NPs. In the previous studies, no obvious dewetting of NPs was observed up to 600 °C and the irregular and networked Pt NPs were demonstrated even at higher temperatures by the SSD of pure Pt films due to the low diffusivity of Pt atoms [31]. On the other hand, the semi-spherical Ag NPs of various sizes with the definite isolation gap were obtained even at ~ 300 °C due to the higher diffusivity of Ag atoms [35]. In the case of Ag/Pt bilayers as shown in Fig. 1a, the dewetting kinetics and the resulting nanostructures can largely be altered upon annealing due to the interplay between Ag and Pt atoms. Initially, the Ag and Pt atoms can inter-diffuse through the Ag/Pt interface via the grain boundaries, triple-junctions, and pinholes, resulting in the increased concentration of Ag in the Pt layer and vice-versa. Consequently, at increased temperature, the Ag and Pt layers can be gradually consumed due to the growth of intermixed region, which can finally transform the Ag/Pt bilayer into a fully alloyed layer as illustrated in Fig. 1b [35–37]. Eventually, the AgPt film starts to agglomerate into the isolated NPs through the void nucleation, growth by the minimization of surface-free energy and interface energy along with the enhanced diffusivity of AgPt alloyed atoms as presented in Fig. 1c [38]. With the further increased temperature, the growth of AgPt alloy NPs can be simultaneously affected by the sublimation of Ag atoms because of the high vapor pressure of Ag, which ultimately leads to the formation of pure Pt NPs due to the extensive desorption of Ag atoms from the AgPt NPs matrix as presented in Fig. 1c, d [39, 40].

The detailed characterization of AgPt alloy and Pt NPs fabricated with the Ag_7nm_/Pt_1.5nm_ bilayers was performed by the morphological and elemental analysis in Fig. 1 and Additional file 1: Figure S2–S3. Upon annealing at 500 °C, the elongated AgPt alloy NPs of ~ 40 nm average diameter (AD) and 20 nm average height (AH) were obtained as displayed in Fig. 1(e), (e-1), and k. As discussed, due to the high diffusivity and low-surface energy of Ag atoms, the intermixing can begin even at a relatively low temperature and thus, the global diffusivity of AgPt alloy can be enhanced and the formation of well-structured and isolated AgPt NPs can be achieved based on the Volmer-Weber growth model [41, 42]. As compared to the previous results of pure Ag or Pt NPs at similar growth conditions, these AgPt NPs showed significant variation in size, configuration, and density, which can be correlated to the enhanced atomic diffusivity of the AgPt alloy system [30, 34]. For instance, these AgPt alloy NPs were generally larger, isolated, and more uniform as compared to those of previously reported pure Pt and Ag NPs, and this clearly suggests an improved surface configuration of AgPt alloy NPs at similar growth conditions. The size of AgPt NPs slightly increased to ~ 42 nm in AD and ~ 10 nm in AH at 550 °C and they were slightly reduced in size at 600 °C as shown in Fig. 11, g, and k. In addition, the evolution of surface NPs was studied in terms of Rq and SAR in Fig. 1l, m, which clearly showed an increased value between 500 °C and 550 °C as then gradual decrement from 600 °C with the variation of AgPt NP’s size. A sharp change in the surface morphology was observed at 650 °C as shown in Fig. 1h, (h-1), and the small and much isolated NPs were obtained and indeed, above 650 °C the size of AgPt alloy NPs were further reduced as seen in Fig. 1i–j.

Along with the increased temperature, the growth of AgPt NPs can be driven by the coalescence growth, surface energy minimization, and equilibrium configuration. Besides, the sublimation of Ag atoms due to its high vapor pressure also can significantly affect the evolution process [39, 40]. The sublimation rate (*R*_*s*_) of Ag can be exponentially increased with the temperature according to the relation: *R*_*s*_ = (3.513 × 1022) (*T M*_Ag_)^−1/2^ × *P*_eq_, where the *T*, *M*_Ag_, and *P*_eq_ are the annealing temperature, molecular weight of Ag, and vapor pressure at *T* [40]. The *P*_eq_ of Ag can reach ~ 1.0 × 10^−7^ Torr at 650 °C, leading to the substantial sublimation rate of Ag atoms [41]. In fact, the sublimation of Ag atoms from the alloy NPs matrix can cause a significant reduction in size and Ag percentage [40]. The EDS analysis was performed to determine the elemental compositions of the alloy NPs and from the EDS spectra and atomic (%) summary plots in Fig. 1n–p and Additional file 1: Figure S3, the variation in Ag and Pt peaks of alloy composition were clearly detected. Specifically, the at % of Ag was sharply attenuated between 500 °C and 650 °C, and the Ag peaks completely vanished as shown in Fig. 1o, p. As the sublimation of Ag atoms was intensified at increased temperature the size of alloy NPs was noticeably reduced above 650 °C as discussed, resulting in pure Pt NPs. At the temperature higher than 650 °C, all the samples had 100% Pt and 0% Ag. From the results, it can also be indicated that the dewetting kinetics of Pt can be enhanced by using Ag layer, which preferentially sublimated at the temperature above 650 °C, resulting in the formation of nearly pure Pt NPs with well-defined and separated configuration [44]. Overall, the AD and AH of Pt NPs were decreased from ~ 44 nm to 23 nm and ~ 12 nm to 7 nm, respectively, when the temperature varied between 550 and 800 °C as summarized in Fig. 1k. Furthermore, the summary plots of Rq and SAR in Fig. 1l, m also showed gradual decrement as the NPs became smaller at higher temperatures, which indicates that the average height and 3D surface area of the NPs were further reduced as discussed above.

**Fig. 2:**
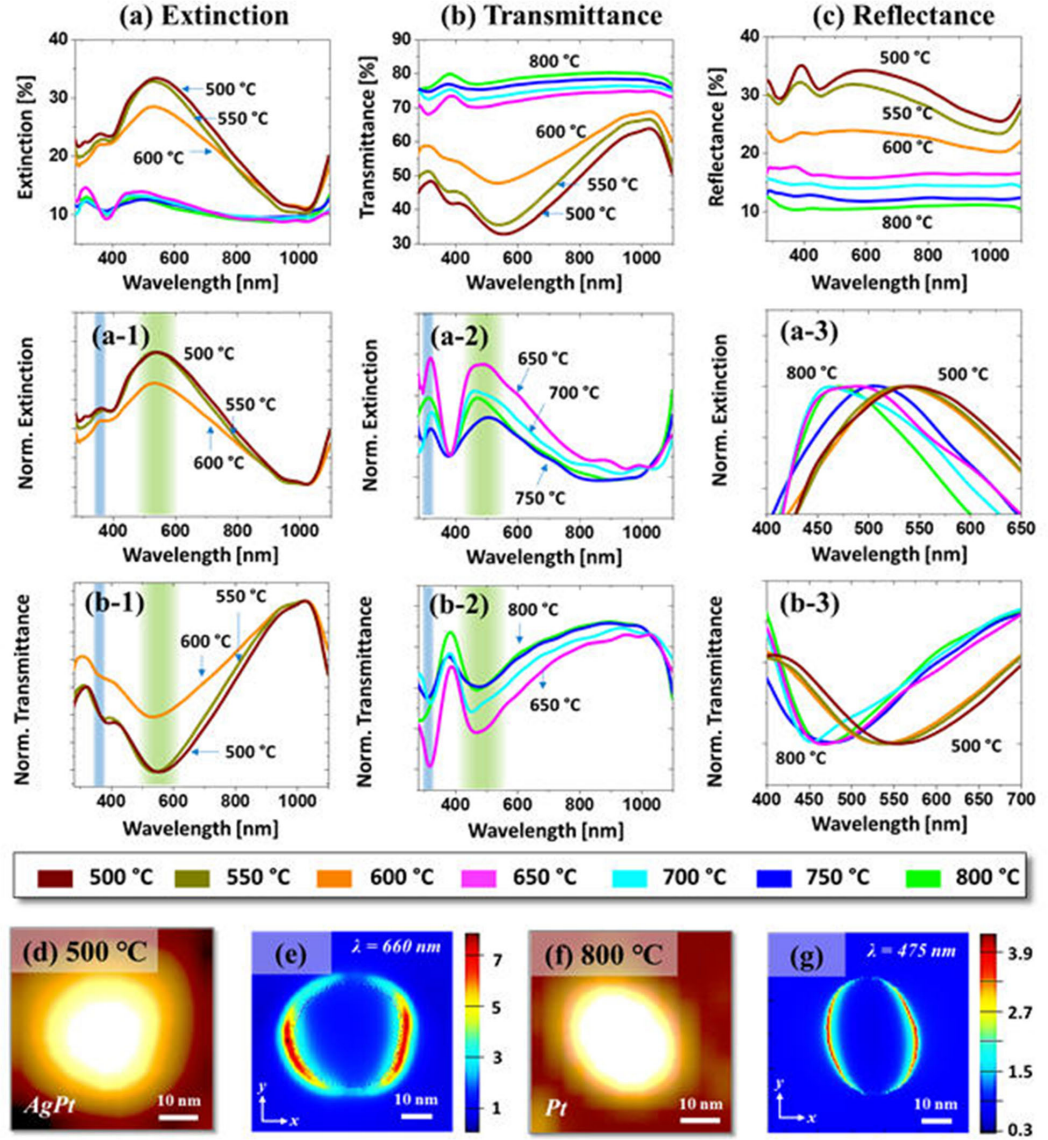
Optical properties of the AgPt and Pt NPs fabricated with the Ag7nm/Pt1.5 nm bilayers. **a**–(**a-3**) Extinction and normalized extinction spectra. **b**–(**b-3**) Transmittance and normalized transmittance spectra. **c** Reflectance spectra. The reflectance and transmittance spectra were acquired from the measurement and the extinction spectra were calculated by the relation: *E* [%] = 100 [%] − (*R* + *T*) [%]. **d, f** AFM images of the typical AgPt and Pt NPs selected for finite-difference time domain (FDTD) simulations. **e, g** Corresponding e-field distribution at resonant wavelength

Figure 2 shows the optical properties of NPs fabricated with the Ag_7nm_/Pt_1.5nm_ in the UV-VIS-NIR region (280–1100 nm). In addition, the local e-field distribution and extinction power spectra of typical AgPt and Pt NPs were simulated by using FDTD solutions. The optical spectra demonstrated various wavelength-dependent LSPR responses based on the structural and elemental variation of AgPt and Pt NPs. Generally, the extinction spectra in Fig. 2a showed two-grouped behaviors for instance, sharply distinct spectral response with the large AgPt NPs up to 600 °C and small Pt NPs above 650 °C. In specific, a strong peak in the VIS and a relatively weak peak in the UV region was developed with the AgPt NPs, which can be due to the excitation of quadrupolar (QR) and dipolar resonance (DR) mode [45]. As shown by the color bands in Fig. 2a–1, the DR and QR bands of AgPt NPs were positioned ~ 540 nm and ~ 365 nm, which showed gradual reduction in intensity at increased temperature likely due to the sublimation of Ag atoms. The e-field distribution on typical AgPt (diameter ~ 40 nm, height ~ 8 nm) NP fabricated at 500 °C is shown in Fig. 2d–e and the extinction power spectrum is presented in Additional file 1: Figure S4(b). In particular, the simulated extinction maxima of AgPt NP was observed at 660 nm due to the dipolar resonance, which was ~ 120 nm red-shifted from the experimental result. This can be due to the size distribution of NPs and the coupling between the plasmon resonance of the individual NPs in the real sample. Nevertheless, the typical AgPt NP showed strong dipolar e-field in the VIS region.

**Fig. 3:**
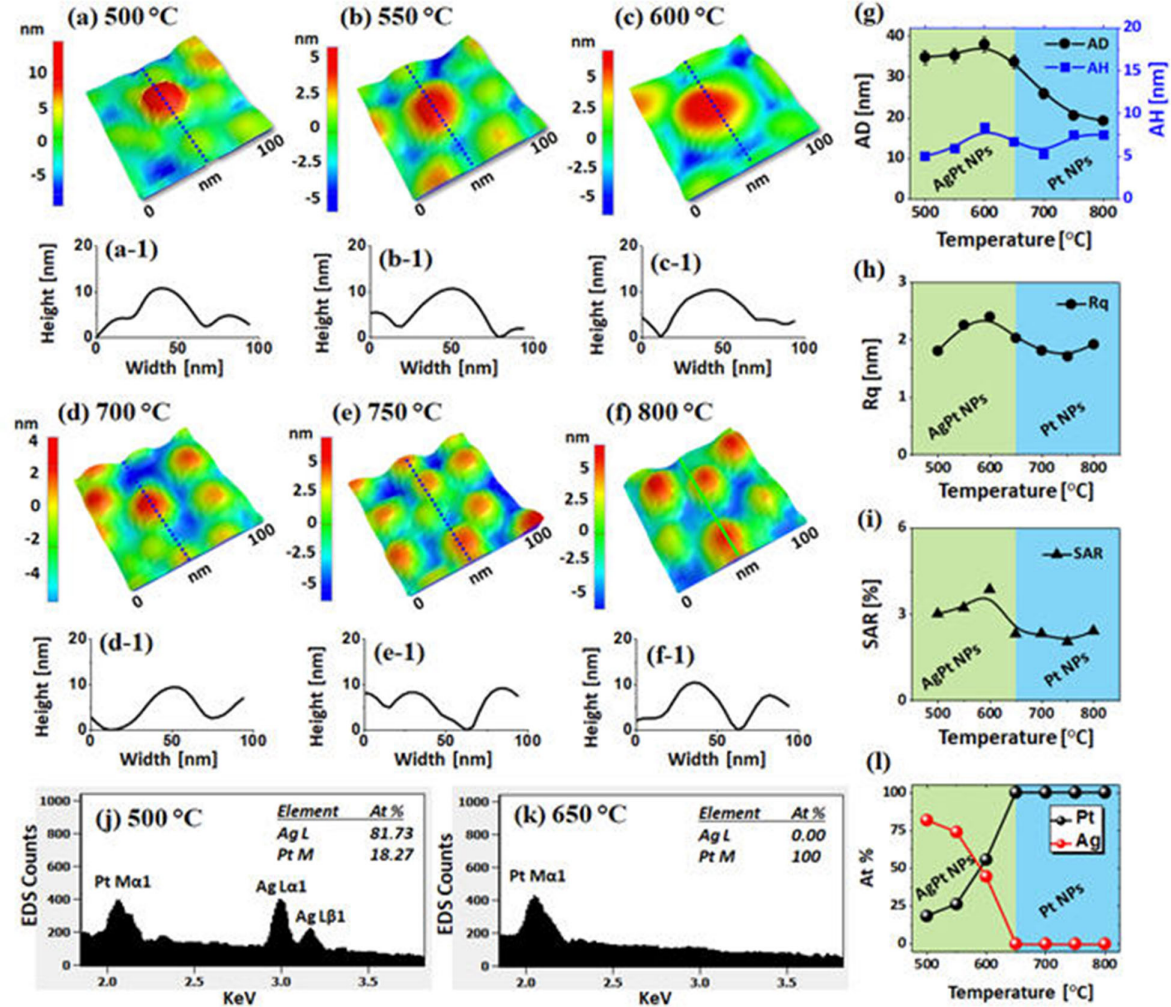
Evolution of AgPt and Pt NPs from the Ag_5nm_/Pt_2.5nm_ bilayers by annealing between 500 °C and 800 °C for 120 s. **a**–**f** AFM side-views. (**a-1**)–(**f-1**) Cross-sectional line profiles. **g**–**i** Summary plots of AD, AH, Rq, and SAR. **j**–**k** EDS spectra of the AgPt and Pt NPs fabricated at 500 and 650 °C. **l** Summary plots of at % of Pt and Ag. The elemental composition of Ag and Pt are enclosed in the EDS spectra

With the formation of Pt NPs at high temperature (> 650 °C), both the DR and QR resonance bands were blue shifted to ~ 460 and 320 nm respectively as shown in Fig. 2(a-2), which can be due to the size reduction of NPs [46, 47]. The overall blue shift trend of DR peaks is clearly traced in Fig. 2(a-3). At the same time, a mild reduction in the DR peak intensity was also observed as can be correlated to the Ag sublimation as well as size reduction. However, the QR peak became comparatively stronger with the small-sized Pt NPs, which can be described by the stronger LSPR effect of Pt in the UV region [48]. Furthermore, the DR peaks were found to be narrowed with the Pt NPs at high temperature likely due to the improved uniformity of NPs. The simulation results of the e-field intensity and extinction power spectrum of typical Pt NPs are shown in Fig. 2f–g and Additional file 1: Figure S4(e)–(h). As compared to the AgPt NPs, the extinction maxima was significantly blue-shifted to ~ 475 nm with relatively small Pt NP, which agrees well with the experimental results. At the same time, the extinction power intensity was also significantly reduced due to the weaker plasmonic effect of pure Pt NP.

In addition, the transmittance spectra in Fig. 2b also showed two-grouped behaviors with the large AgPt and small Pt NPs. The larger AgPt NPs up to 600 °C featured a minor dip in the UV region at ~ 365 nm and a strong dip in the visible region ~ 540 nm corresponding to the QR and DR band as indicated by the color band in Fig. 2(b-1) [45]. With the sublimation of Ag at increased temperature, the DR bands were sharply reduced as displayed in Fig. 2(b-1) indicating lower absorption with Ag sublimation. However, the QR bands of small Pt were found to be relatively stronger as compared to that of large AgPt NPs as shown in Fig. 2(b-2). Since the average size of Pt NPs was slightly decreased with temperature, the absorption dips were also mildly reduced or similar. At the same time, the obvious blue shift of QR and DR bands to ~ 320 and 460 nm, respectively, was observed as the overall size of Pt NPs was reduced [47, 48]. The position of DR band was traced in Fig. 2(b-3), which also showed the blue shift from ~ 540 nm to ~ 460 nm for the NPs fabricated between 500 °C and 800 °C.

In terms of reflectance analysis as displayed in Fig. 2c, again two-grouped behaviors appeared. With AgPt NPs below 600 °C, the spectra were generally comprised of dips in the UV and VIS regions followed by the strong shoulder between ~ 500 and 1000 nm; however, the reflectance spectra did not clearly exhibit the absorption dips but rather showed the strong shoulders. This can be caused by the strong backscattering effect of AgPt NPs [49]. As the size of AgPt NPs and Ag atoms were reduced with temperature, the backscattering shoulder were gradually attenuated and finally became flat with the Pt NPs as shown in Fig. 2c. From the overall optical spectra analysis, the AgPt NPs generally exhibited a much stronger LSPR band in the VIS region with the high percentage of Ag component. However, even with the near-pure Pt NPs at high temperature, the LSPR peaks were still considerably strong. As compared to the weak and broad LSPR band in the VIS region with the irregular and random Pt NPs from the previous study, the LSPR properties were greatly enhanced because of the improvement in size, uniformity, and elemental combination with Ag [31]. Furthermore, the LPSR peaks in terms of intensity and peak positions were found to be more dynamic and sensitive to the evolution of AgPt and Pt NPs, which can be attributed to the size and elemental composition variation along with the enhanced dewetting process.

Figure 3 shows the fabrication of AgPt and Pt NPs with the Ag_5nm_/Pt_2.5nm_ bilayer series annealed between 500 and 800 °C for 120 s. In this set, the thickness of the Pt top-layer was increased whereas the Ag underlayer was decreased. As a result, the dewetting process can be slightly hindered as the portion of high-diffusivity material was decreased whereas the low-diffusivity material was increased [37]. However, due to the atomic inter-diffusion and intermixing between Ag and Pt layers, the overall dewetting can still be enhanced as compared to the pure Pt layers [31]. The surface morphology of NPs at different temperatures can be observed in Fig. 3a–f. Furthermore, the large-scale AFM images are provided in Additional file 1: Figure S4–S6. As displayed by the AFM images, the NPs were generally smaller and denser with the semi-spherical configurations as compared with the previous set likely due to the slightly hindered diffusion. More specifically, at 500 °C, the isolated but slightly irregular AgPt NPs of 35 nm AD and 10 nm AH were obtained.

By comparing with the previous set, these NPs were slightly smaller and more irregular at this temperature, which can be again correlated to the hindered diffusivity as discussed. With the increment of temperature up to 600 °C, the size and spacing of alloy NPs were gradually increased due to the enhanced diffusion as well as coalescence growth of neighboring NPs, i.e., the AH and AD were increased to ~ 40 and 20 nm, respectively, as shown in Fig. 3g. As the temperature was increased above 600 °C, the AD of AgPt NPs was gradually decreased whereas the AH remained similar. The size reduction at high temperature can be due to the sublimation of Ag atoms as discussed, which was confirmed by the EDS analysis as presented in Figs. 3j–l. The at % of Ag atoms were sharply reduced between 500 °C and 600 °C and became zero at higher temperature. This again clearly indicated the formation of near pure Pt NPs above 650 °C.

The size evolution of NPs was further studied by the Rq and SAR as shown in Fig. 3h, i, respectively. Both the Rq and SAR were slightly increased up to 600 °C due to the growth of NPs size. However, as the Ag sublimation was intensified at a high temperature, the NPs were again reduced in size. Meanwhile, the shape of NPs became more spherical with the improved uniformity as NPs could possess an equilibrium configuration with the minimum surface energy. Again, these results clearly demonstrated a significant improvement in the size, shape, and uniformity of AgPt NPs in comparison with the pure NPs of parent elements from the previous studies, which can be attributed to the enhanced diffusion with the AgPt alloy system [31, 35].

**Fig. 4:**
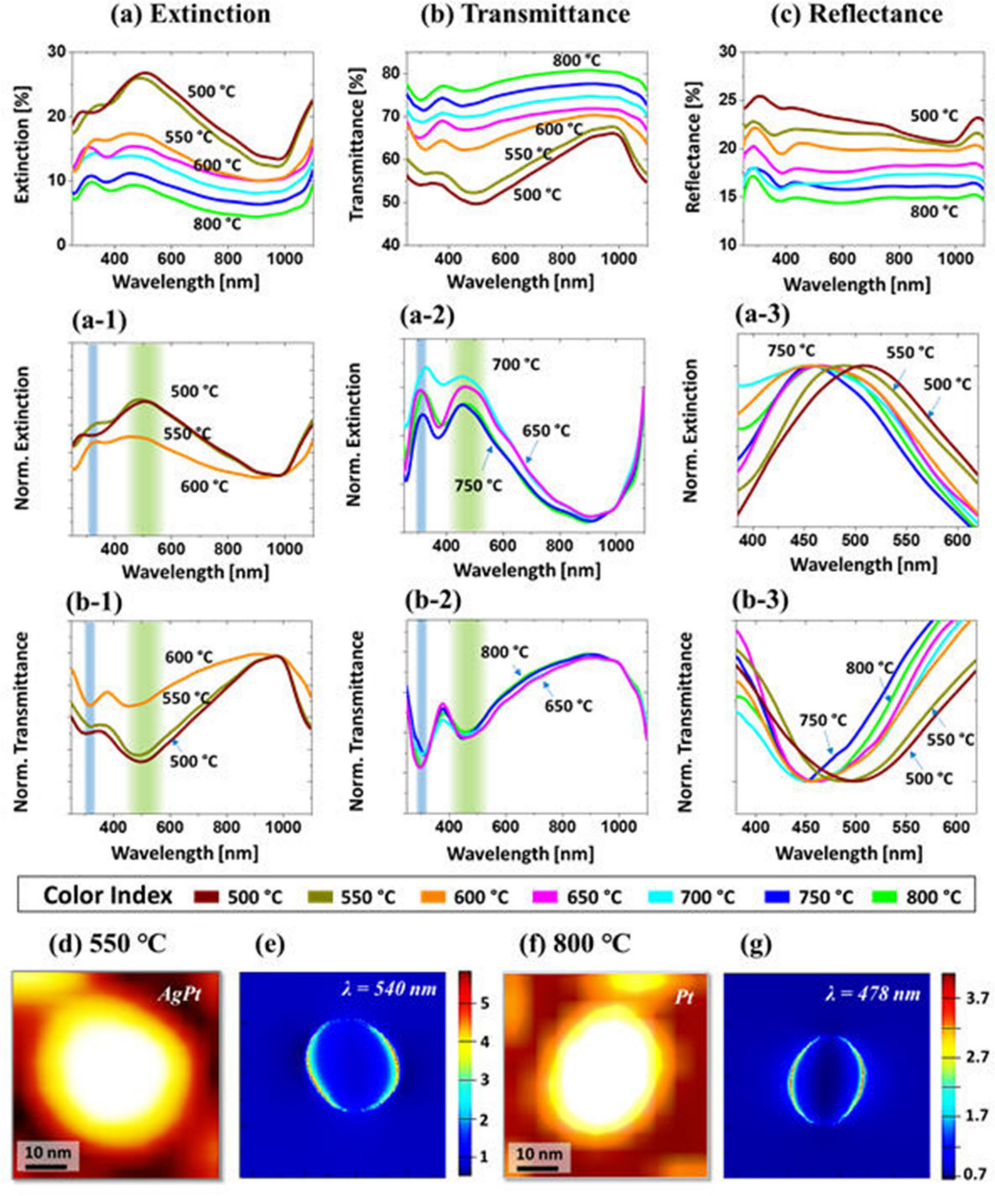
LSPR properties of the AgPt and Pt NPs fabricated with the Ag5nm/Pt2.5 nm bilayers by annealing between 500 and 800 °C. **a**–(**a-3**) Extinction and normalized extinction spectra. **b**–(**b-3**) Transmittance and normalized transmittance spectra. **c** Reflectance spectra. **d, f** AFM images of the typical AgPt and Pt NPs selected for FDTD simulation. **e, f** E-field distribution in *xy*-plane

Figure 4 presents the optical properties of AgPt and Pt NPs fabricated with the Ag_5nm_/Pt_2.5nm_ bilayers between 500 °C and 800 °C. In comparison with the previous set, the optical properties in terms of LSPR band positions and intensity were readily varied due to the difference in size and elemental composition of NPs. Basically, this set of samples also showed the distinct optical behaviors with the relatively larger AgPt NPs up to 600 °C and smaller Pt NPs above 650 °C as in the previous set. As shown in Figs. 4a, (a-1), the extinction spectra exhibited a DR in the VIS at ~ 500 nm and a QR in the UV at ~ 340 nm with the AgPt NPs. These LSPR peaks were found to be comparatively weaker, and blue-shifted from the previous set, which can be due to the less Ag content and smaller size of AgPt NPs [43, 44]. Similar to the previous set, the LSPR intensity was gradually attenuated along with the sublimation of Ag at increased temperature although the average size of NPs was slightly increased as shown in Fig. 4(a-1). From the simulation of the e-field and extinction power spectrum of the typical AgPt NP, it also exhibited a strong DR at ~ 540 nm as shown in Fig. 4d, e and Additional file 1: Figure S8(a)–(d). As seen in Fig. 4e, the e-field was strongly confined at the boundary of the alloy NPs due to the DR effect. As the smaller Pt NPs were formed at high temperature, the DR and QR peaks were further blue-shifted to ~ 460 and 320 nm respectively as presented in Fig. 4(a-2). The overall shift of DR peak is presented in Fig. 4(a-3), which showed the gradual blue shift of peak position from ~ 500 nm to 460 nm with the reduction of NPs size. At the same time, while the DR peaks were significantly attenuated, the QR peaks were slightly enhanced than that of AgPt NPs, which can be likely due to the strong LSPR response of Pt in the UV region as discussed [48]. However, due to the mild evolution of Pt NPs at a higher temperature, the LSPR peak intensity was not changed significantly. In addition, the e-field distribution of typical Pt NP is presented in Fig. 4d–f, which clearly exhibited a DR at 478 nm. The corresponding extinction power spectrum is provided in Additional file 1: Figure S8(f), whose maximum was shifted to 478 nm and intensity was decreased by two-fold as compared to the AgPt NP.

The LSPR characteristic of AgPt and Pt NPs at different resonance bands were also demonstrated by the transmittance spectra in Fig. 4b–(b-3). In specific, the QR and DR dips were observed at ~ 330 nm and 500 nm, respectively, which also showed blue shift in comparison with the previous set due to the smaller size of AgPt NPs. Furthermore, the absorption dip intensity was gradually reduced at high temperature along with the sublimation of Ag as well as NPs size reduction as discussed [47]. From the transmittance dips also, the UV absorption was found to be enhanced with the Pt NPs as shown in Fig. 4(b-2). The position of DR dip was traced in Fig. 4(b-3), which showed the blue shift from ~ 500 to 450 nm with the NPs fabricated between 500 and 800 °C. In terms of the reflectance spectra in Fig. 4c, it exhibited a narrow shoulder in the UV at ~ 300 nm and flat shoulder in the VIS region. In contrast to the previous set, the UV shoulder was blue-shifted, and the VIS shoulder was significantly attenuated. As the AgPt NPs were slightly smaller in this set, the enhanced backscattering can be expected with the strong DR mode; however, indeed, less backscattering with the flatter VIS spectra was observed, which can be likely due to the reduced Ag percentage in the AgPt NPs. Nevertheless, the absorption dips were buried by the backscattering as seen in the reflectance spectra [49]. Along with the sublimation of Ag and formation of smaller Pt NPs at increased temperature, the UV-VIS portion was gradually attenuated indicating further suppression of the backscattering effect. From all the optical spectra of AgPt and Pt NPs, the LSPR peaks were generally found in the shorter wavelength and relatively attenuated due to the smaller size and lower Ag content as compared to the previous set while it still demonstrated the improved as compared to the Pt NPs from the previous study [31].

**Fig. 5:**
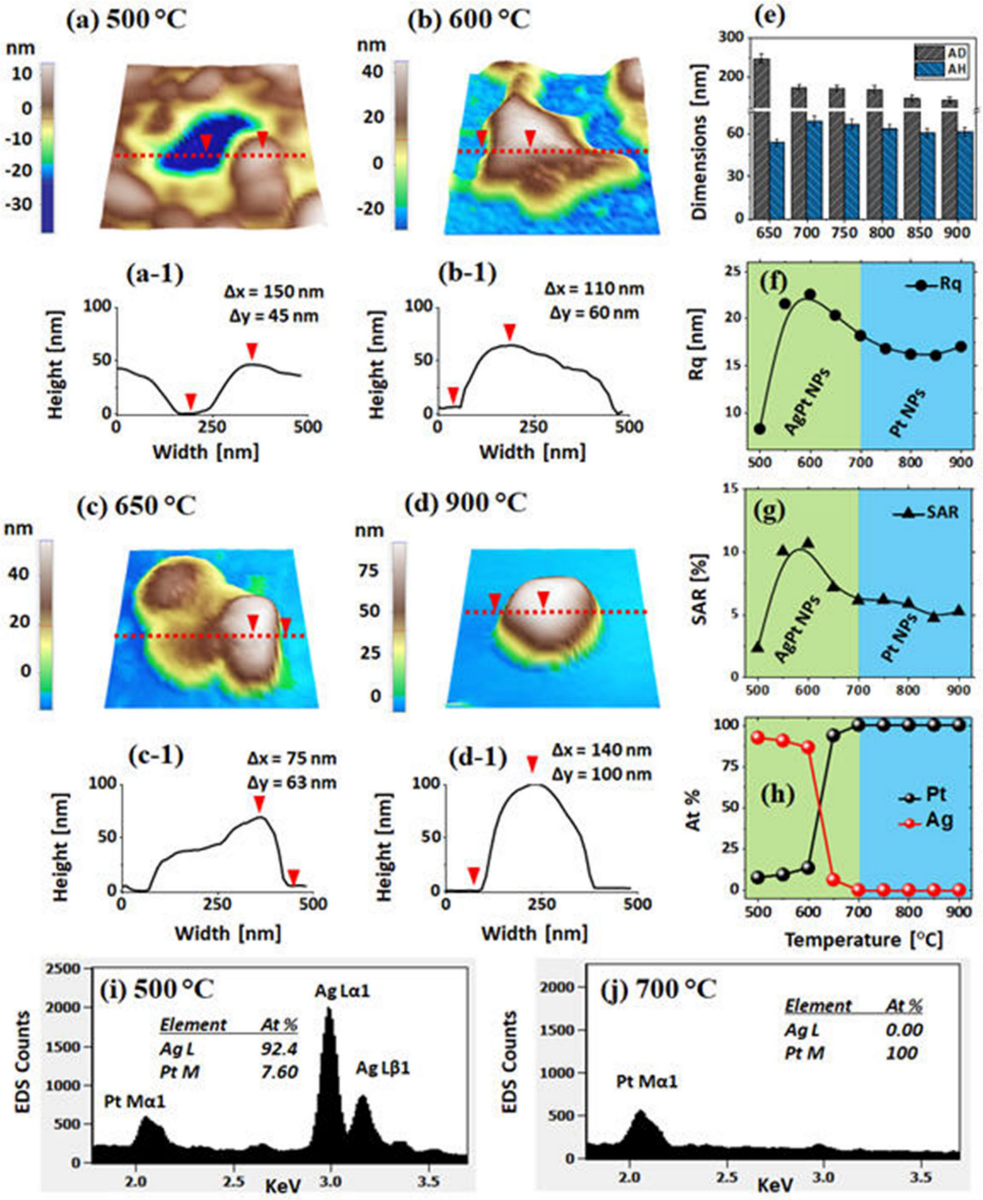
Evolution of AgPt nanoclusters and Pt NPs by the dewetting of Ag_21nm_/Pt_4.5nm_ bilayers between 500 °C and 900 °C for 120 s. **a**–**d** AFM side-views of 500 × 500 nm^2^. (**a-1**)–(**d-1**) Cross-sectional line profiles. **e** Summary plots of AD and AH of isolated NPs above 650 °C. **f, g** Summary plots of Rq and SAR. **h** Plot of at % of Ag and Pt at increased temperatures. **i**, **j** EDS spectra of the samples annealed at 500 and 700 °C. The elemental composition of Ag and Pt are enclosed in the EDS spectra

**Fig. 6:**
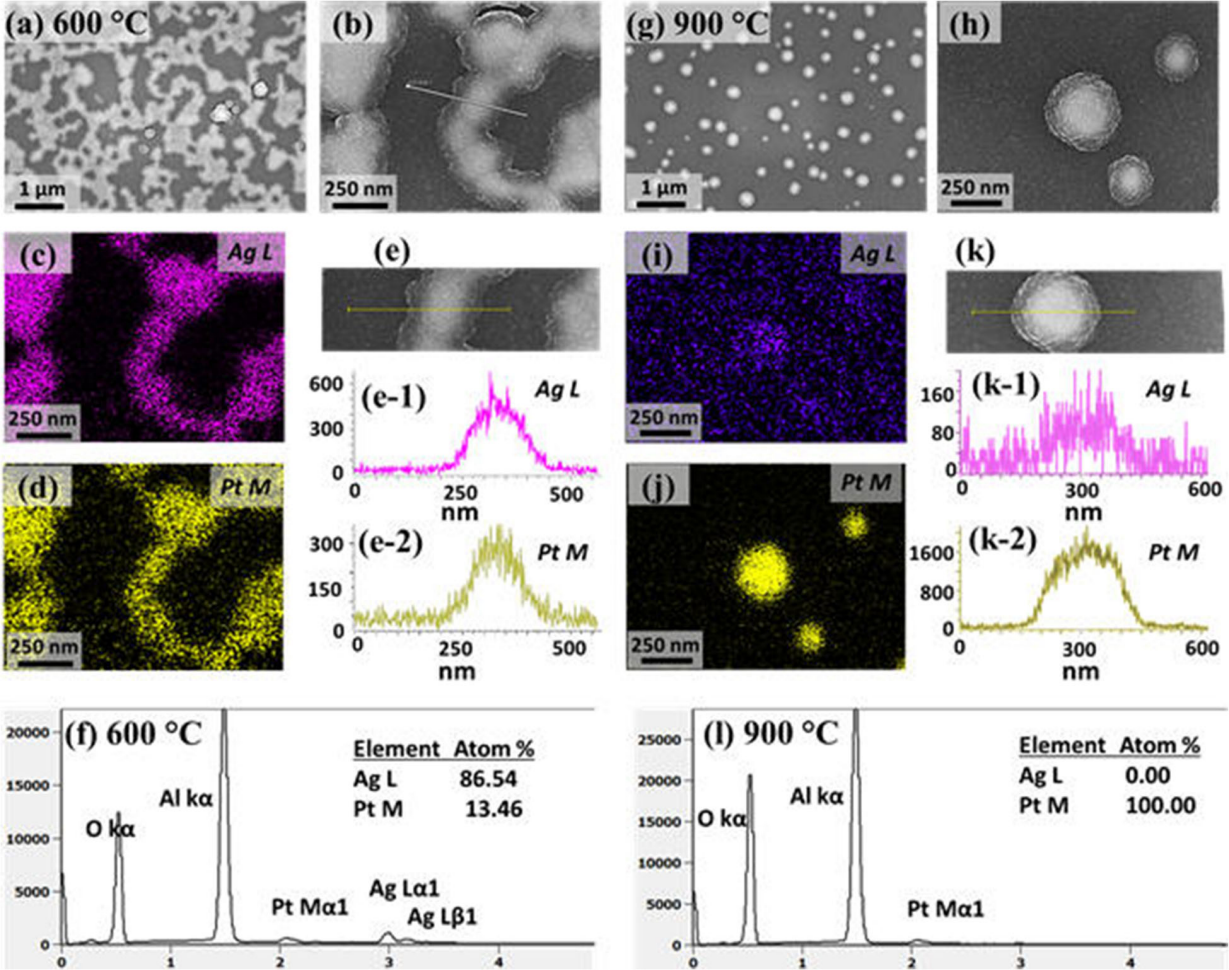
Elemental analysis of the typical AgPt and Pt NPs fabricated with the Ag21nm/Pt4.5 nm bilayers at 600 and 900 °C for 120 s. **a, b** SEM images of AgPt alloy nanoclusters. **c**, **d** Elemental phase map of Ag L and Pt M. **e**–(**e-2**) Elemental line profiles of Ag and Pt through the nanocluster. **f** EDS spectra of the AgPt alloy nanoclusters at 600 °C. **g**, **h** SEM images of Pt NPs. **i**, **j** Elemental phase maps. **k**–(**k-2**) EDS line profile through the typical Pt NP. **l** EDS spectra of Pt NPs at 900 °C. The EDS map, line profile, and spectra show the absence of Ag in the NPs at 900 °C due to the sublimation

Figure 5 presents the fabrication of large AgPt and Pt NPs by the dewetting of thicker Ag_21nm_/Pt_4.5nm_ bilayers at an identical growth condition as in the previous sets. The growth characteristics of NPs were drastically varied in terms of the size, shape, and density due to the added thickness of Ag and Pt layers. In specific, the wide coverage large AgPt nanoclusters below 650 °C and widely spaced semi-spherical Pt NPs above 650 °C were obtained as shown by the AFM images. Since the dewetting of thin films depends on the initial thickness, the final nanostructures can also vary with the thickness [35]. Generally, the thinner films yield the small and dense NPs, and the thicker films produce the widely spaced large NPs. As the larger NPs are formed, the surface chemical potential becomes much lower at the NP sites and thus adatom diffusion and absorption boundary are significantly enhanced, thus yielding much widely spaced large NPs [50, 51]. Meanwhile, the temperature required to obtain the definite structure also increases with the thickness due to the increased thermal stability of thicker films. As shown in Fig. 5a, only the nucleation of voids and grains was observed at 500 °C, and the diameter and height of nanovoids were about 300 and 45 nm respectively. As the temperature was increased, the voids were expanded whereas the nanoclusters were agglomerated more compactly to form the irregular nanoclusters up to 600 °C. This resulted in the large reduction of surface coverage and the nanostructures were grown in 3D form based on the Volmer-Weber growth model [41]. The large-scale AFM and SEM images at specific temperatures are provided in Additional file 1: Figure S8–S13. In addition, the detailed elemental analysis is performed with the sample annealed at 600 °C as shown in Fig. 6a–f. As the dewetting advances with temperature, the interconnected AgPt alloy nanoclusters were developed as shown by SEM images in Fig. 6a–b. The corresponding elemental phase of Ag L and Pt M in Fig. 6c, d matched well with the SEM images. Furthermore, the EDS line profile through the nanoclusters clearly revealed the homogeneous distribution of Ag and Pt atoms as shown in Fig. 6e. The elemental composition was also substantially altered along with the temperature as clearly shown in Fig. 5i, f in which the at % of Ag was decreased from 92.4 to 86.54% when temperature rise from 500 °C to 600 °C.

A sharp change in the surface morphology was observed when the connected nanoclusters were broken into the isolated-irregular NPs at 650 °C due to the Rayleigh-like instability of large nanoclusters [49] as shown in Fig. 5c. The widely isolated NPs above 650 °C were gradually reduced in size and became more spherical. The AH and AD of NPs were decreased from ~ 250 nm to 180 nm and ~ 80 nm to 60 nm, respectively, as displayed in Fig. 5e. Around this temperature, the Ag sublimation was also extensive, which can result in almost pure Pt NPs. The EDS at % of elements and EDS spectra in Fig. 5h–j depict the large desorption of Ag atoms between 600 and 650 °C. Specifically, with the formation of isolated NPs above 700 °C, the at % of Ag became zero and Ag peak disappeared, indicating no Ag content in the surface of samples [43]. Therefore, the size of NPs was noticeably reduced above 650 °C and the NPs can be of near pure Pt. Finally, the semi-spherical and widely isolated Pt NPs were resulted by increasing temperature up to 900 °C as displayed in Fig. 5d. The detailed elemental analysis of NPs at 900 °C clearly revealed the absence of Ag atoms while the overall NP is constituted by the Pt atoms as shown in Fig. 6g–l. As discussed, the overall transformation of AgPt and Pt NPs can be simultaneously governed by the energy minimization and Ag sublimation. By comparing with the previous results of thinner Ag and Pt films, although the dewetting sequence was similar, the surface configuration, size, and spacing of AgPt NPs were significantly altered due to the enhancement in the dewetting degree [31, 35]. For instance, the isolated Pt NPs possessed much wider spacing and lower density but larger size than Ag and Pt NPs from the previous reports. In addition, the SAR and Rq summaries in Fig. 5f, g showed the increasing values from ~ 2 to 11% and ~ 8 to 23 nm, respectively, up to 600 °C along with the evolution of voids and 3D nanoclusters. Then, the SAR and Rq were gradually decreased as the nanostructures were fragmented and isolated along with the temperature. The surface morphology as well as the elemental composition of AgPt NPs has simultaneously changed due to the dewetting and sublimation as in the previous sets.

**Fig. 7:**
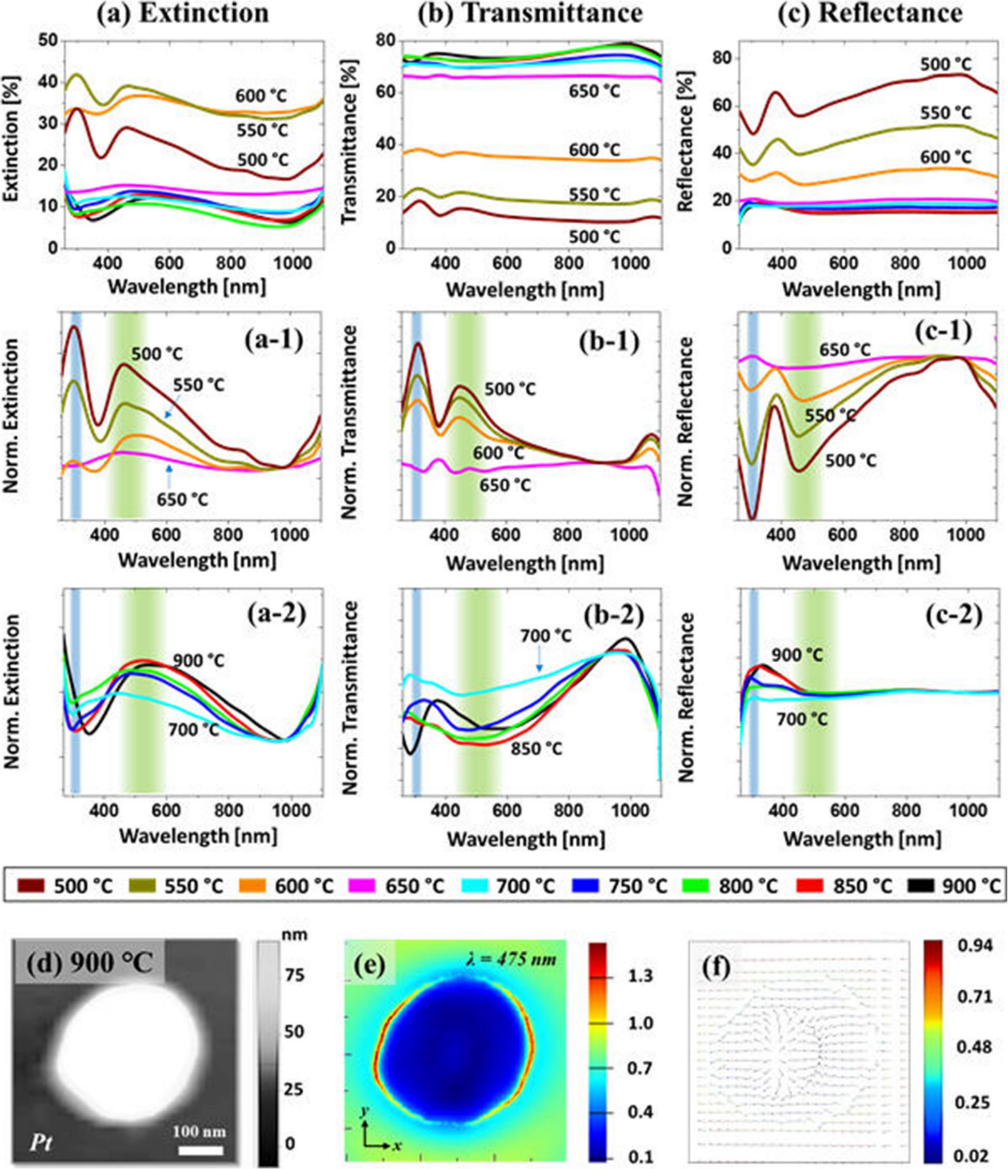
LSPR properties of the AgPt nanoclusters and Pt NPs fabricated with the Ag21nm/Pt4.5 nm bilayer between 500 and 900 °C. **a**–(**a-2**) Extinction and normalized extinction spectra. **b**–(**b-2**) Transmittance and normalized transmittance spectra. **c**–(**c-2**) Reflectance and normalized reflectance spectra. **d** AFM image of the Pt NP selected for the FDTD simulations. **e** E-field distribution. **f** Corresponding e-field vector profile

Figure 7 shows the optical properties of large AgPt and Pt NPs on sapphire fabricated with the Ag_21nm_/Pt_4.5nm_ bilayer at various temperatures. Apparently, the optical properties of large AgPt and Pt NPs were drastically varied in terms of spectral shape as compared to the small NPs in the previous sets. The overall optical spectra in this set also can be divided into two groups based on the spectral response of wide coverage large AgPt nanoclusters up to 650 °C and the isolated spherical Pt NPs above 650 °C. For instance, the extinction spectra in Fig. 7a, (a-1) demonstrated two strong peaks in the UV region at ~ 300 nm and in the VIS region at ~ 460 nm with the large and wide coverage AgPt NPs. As the AgPt nanostructures were generally large with wide coverage and size distribution, the LSPR peaks can be mainly contributed by the multipolar (MR) and higher-order resonance (HR) modes in the VIS and UV regions respectively [44]. Since the AgPt nanoclusters were generally larger, the multipolar resonance can more significantly contribute to the VIS absorption band and the LSPR peaks were gradually reduced and became broader with the sublimation of Ag at high temperature as shown in Fig. 7(a-1) [47, 48].

The isolated and relatively smaller Pt NPs at higher temperature exhibited a much wider peak in the VIS region (450–600 nm) and a minor shoulder in the UV region (~ 300–310 nm) as shown in Fig. 7(a-2). In this case, the MR band in the VIS region can be mainly contributed by the multiple DRs and QRs as the size of NPs were significantly increased (200 nm in AD and ~ 60 nm in AH) and other resonance modes can also be present. Because of the overlapping of different resonance modes, the VIS peak can become broader and the peak maximum was found to be in the longer wavelength as compared to the large AgPt nanoclusters. Furthermore, the MR in the VIS wavelength was found to be enhanced with more definite and regular NPs at high temperature as shown in Fig. 7(a-2). The typical large and isolated Pt NP was simulated as shown in Figs. 7d–f and Additional file 1: Figure S14, which showed the strong local e-field enhancement at the edges of NPs due to the MR and HO resonance. In addition, the simulated extinction power spectra exhibited shoulder in the UV and Vis region, which could be due to the overlapping of the various resonance modes in the VIS region and HO modes in the UV regions as discussed. The e-field vector plot was also extracted at ~ 475 nm corresponding to the peak of the VIS shoulder in Fig. 7f depicting multiple directions across the NPs surface. This can imply that the LSPR in the large Pt NP was mostly contributed by the superimposition of MP and HO resonance modes.

Similarly, the transmittance spectra as shown in Fig. 7b, (b-2) showed two distinct behaviors for the wide coverage of large AgPt nanoclusters and isolated semi-spherical Pt NPs. As shown in Fig. 7(b-1), the large AgPt NPs with the high Ag percentage showed the peaks in the UV and VIS region, which can be due to the pronounced forward scattering with the relatively strong QR mode of large AgPt nanoclusters as discussed [45]. With the evolution of isolated and semispherical Pt NPs, the forward scattering was diminished whereas obvious absorption dip in the VIS region was observed as shown in Fig. 7(b-2) as can be correlated to the stronger DR with the smaller size of Pt NPs. From the reflectance spectra in Fig. 7c, (c-1), it exhibited a narrow dip in the UV at ~ 300 nm and a wide dip in the VIS region at ~ 480 nm, corresponding to the HR and MR resonance of the AgPt nanoclusters. The absorption dips were gradually weakened along with the Ag sublimation as displayed in the normalized spectra in Fig. 7(c-1). And when the isolated spherical Pt NPs along with the extensive sublimation of Ag were formed, the reflectance behavior was also readily changed as the UV and VIS dips were disappeared whereas the shoulder started to develop as displayed in Fig. 7(c-2). As the Pt NPs were relatively smaller and isolated, the DR excitation can be expected to be stronger due to which the backscattering effect can be increased, resulting in the shoulder in the UV-VIS region [49].

## Conclusions

In summary, the AgPt and Pt NPs with various sizes and configurations have been demonstrated on sapphire (0001) based on the solid-state dewetting of the three series of Ag/Pt bilayers. By the systematic control of Ag/Pt bilayer thickness and annealing temperature, the semi-spherical AgPt and Pt NPs with the wide range of size and elemental variation were achieved. The dewetting of Ag/Pt bilayer was attributed to the intermixing between Ag and Pt atoms, AgPt alloy formation and enhanced global diffusion of atoms. The elemental composition of AgPt NPs was found to be significantly altered due to the pronounced Ag sublimation above 650 °C, which led to the formation of near pure Pt NPs at higher temperatures. A sharp contrast with the monometallic Pt NPs from the previous studies was presented in terms of morphological and optical property improvement. The optical analysis of AgPt and Pt NPs showed the formation of strong LSPR bands in the UV-VIS region. The morphological improvement was also reflected in the optical properties as the LSPR responses were generally stronger and dynamic as compared to the previous reports. Specifically, the strong LSPR occurred in the VIS region, whose intensity and position were readily altered along with the size and elemental composition of the NPs. Finally, these results demonstrated the wide range of potentials of the structurally and elementally tunable AgPt and Pt NPs by the one-step growth, which could be applicable in the plasmonic-based catalytic and optoelectronic applications.

## Supplementary information

**Additional file 1: Figure S1–S14.** Supplementary materials include the additional AFM and SEM images, EDS spectra and maps, and FTTD simulations of various AgPt and Pt NPs. The datasets used and/or analyzed during the current study are available from the corresponding author upon a reasonable request. **Table S1-S2.** Summary of geomatical values including Rq, SAR, AH and AD.

## Data Availability

Additional file 1: Figures S1-S14 includes the additional AFM and SEM images, EDS spectra and maps, and FTTD simulations of various AgPt and Pt NPs. Table S1 - S2 Summary of geomatical values including Rq, SAR, AH and AD.The datasets used and/or analyzed during the current study are available from the corresponding author upon a reasonable request.

## Abbreviations

AD: Average diameter
AFM: Atomic force microscope
Ag: Silver
AH: Average height
at%: Atomic percentage
Au: Gold
DR: Dipolar resonance
EDS: Energy-dispersive X-ray spectroscope
FDTD: Finite-difference time domain
HR: Higher-order resonance
LSPR: Localized surface plasmon resonance
MR: Multipolar resonance
NIR: Near-infrared
NPs: Nanoparticles
PLD: Pulsed laser deposition
PML: Perfectly matched layer
Pt: Platinum
QR: Quadrupolar resonance
Rq: RMS roughness
SAR: Surface area ratio
SEM: Scanning electron microscope
SSD: Solid-state dewetting
UV: Ultraviolet
VIS: Visible
