# Supplementary material for: Improved Morphological and Localized Surface Plasmon Resonance (LSPR) Properties of Fully Alloyed Bimetallic AgPt and Monometallic Pt NPs Via the One-Step Solid-State Dewetting (SSD) of the Ag/Pt Bilayers
Source: Nanoscale Res Lett. 2019 Oct 24;14:332. doi: 10.1186/s11671-019-3170-0 (PMC6813404; doi:10.1186/s11671-019-3170-0)
Supplement: Supplementary file 1 — Additional file 1: Figure S1–S14. Supplementary materials include the additional AFM and SEM images, EDS spectra and maps, and FTTD simulations of various AgPt and Pt NPs. The datasets used and/or analyzed during the current study are available from the corresponding author upon a reasonable request. Table S1-S2. Summary of geomatical values including Rq, SAR, AH and AD. [file 11671_2019_3170_MOESM1_ESM.docx]

**Supplementary Information**

***Improved morphological and localized surface plasmon resonance (LSPR) properties of fully alloyed bimetallic AgPt and monometallic Pt NPs via the one-step solid state dewetting (SSD) of the Ag/Pt bilayers***

Sundar Kunwar^1^, Puran Pandey^1^, Sanchaya Pandit^1^, Mao Sui^1,2^ and Jihoon Lee^1*^

^1^ Department of Electronic Engineering, College of Electronics and Information, Kwangwoon University, Nowon-gu Seoul 01897, South Korea.

^2^ Institute of Hybrid Materials, College of Materials Science and Engineering, Qingdao University, Qingdao 266071, P. R. China

*Correspondence e-mail: jihoonleenano@gmail.com

**
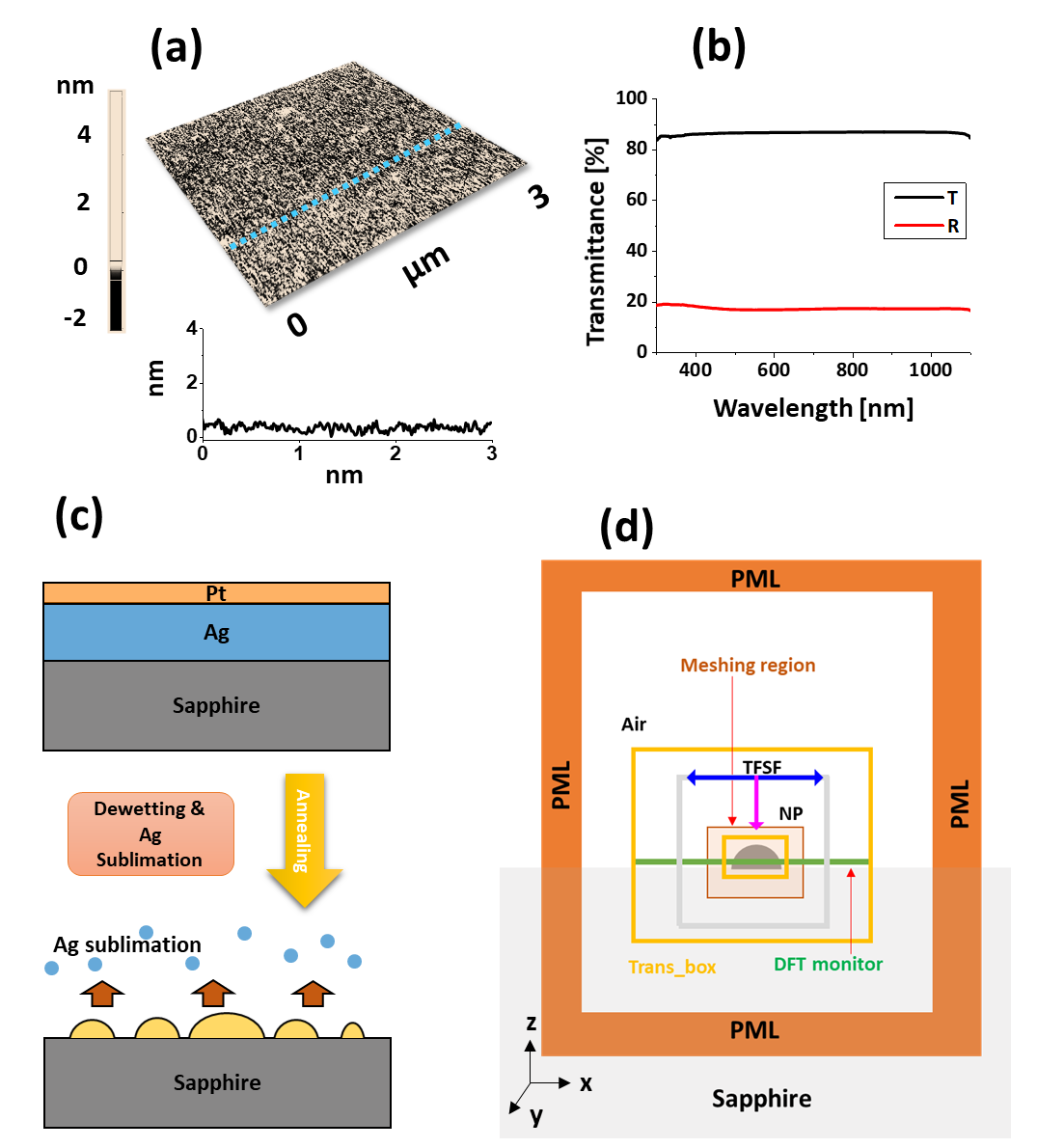
**

**Figure S1:** (a) Atomic force microscope (AFM) side-views of bare sapphire (0001) and cross-sectional line profile. (b) Transmittance and reflectance spectra between 200 and 300 nm of bare sapphire. (c) Schematic of Ag/Pt bilayer deposition on sapphire (0001) and dewetting of the AgPt and Pt NPs. (d) Schematic diagram of the finite difference time domain (FDTD) simulation setup. The typical AFM images of surface nanostructures were imported in to the object space. The TFSF source was engaged from z-direction to excite the sample. The trans-box monitors were used inside and outside of the total field scattered field (TFSF) source to determine the absorption and scattering power respectively. Finally, the extinction power is achieved by adding the absorption and scattering power. The perfectly matched layer (PML) boundary condition was applied in x,y and z-directions. The 3D mesh grid of 0.05 to 0.5 nm was applied around the nanostructures.

**
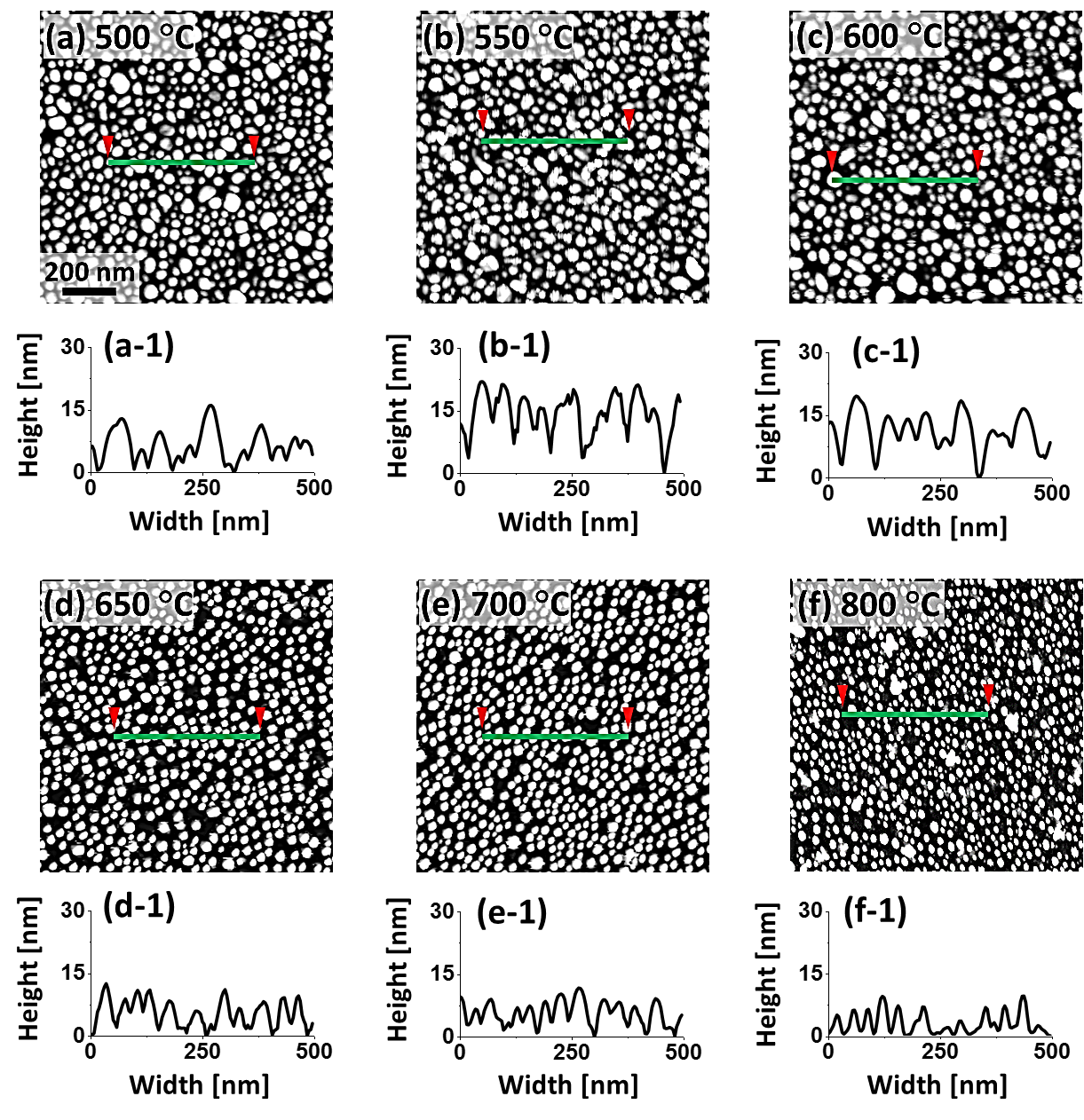
**

**Figure S2:** Evolution of AgPt and Pt NPs from the Ag_7nm_/Pt_1.5nm_ bilayers by annealing between 500 and 800 °C for 120 s. (a) – (f) AFM top-views of 1 × 1 μm^2^. (a-1) – (f-1) Cross-sectional line profiles based on the line in AFM top-views.

**
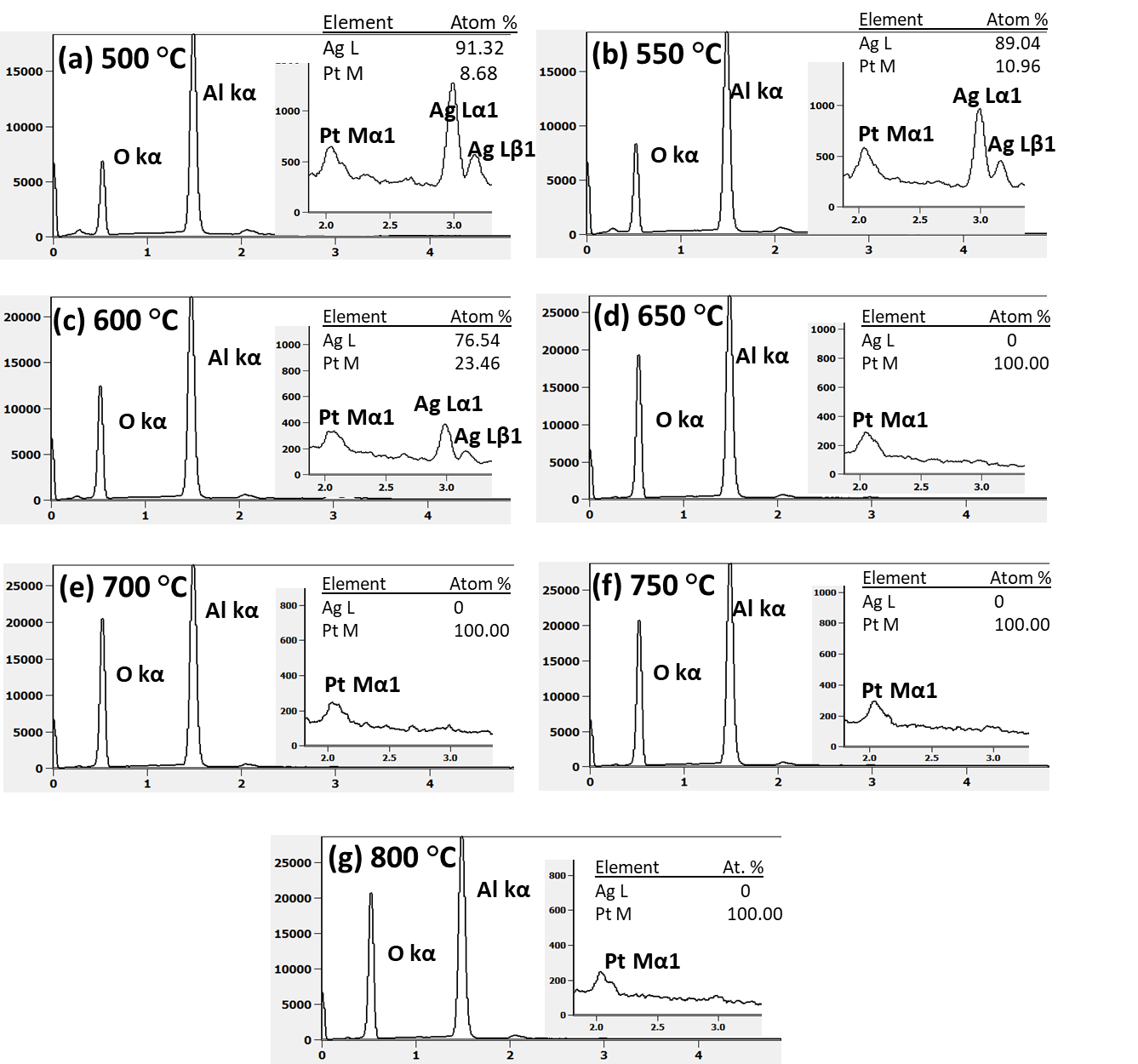
**

**Figure S3:** EDS spectra of the AgPt and Pt NPs fabricated on sapphire with the Ag_7nm_/Pt_1.5nm_ bilayers by annealing between 500 and 900 °C for 120 s. Corresponding insets show the enlarged Pt and Ag peaks.

**
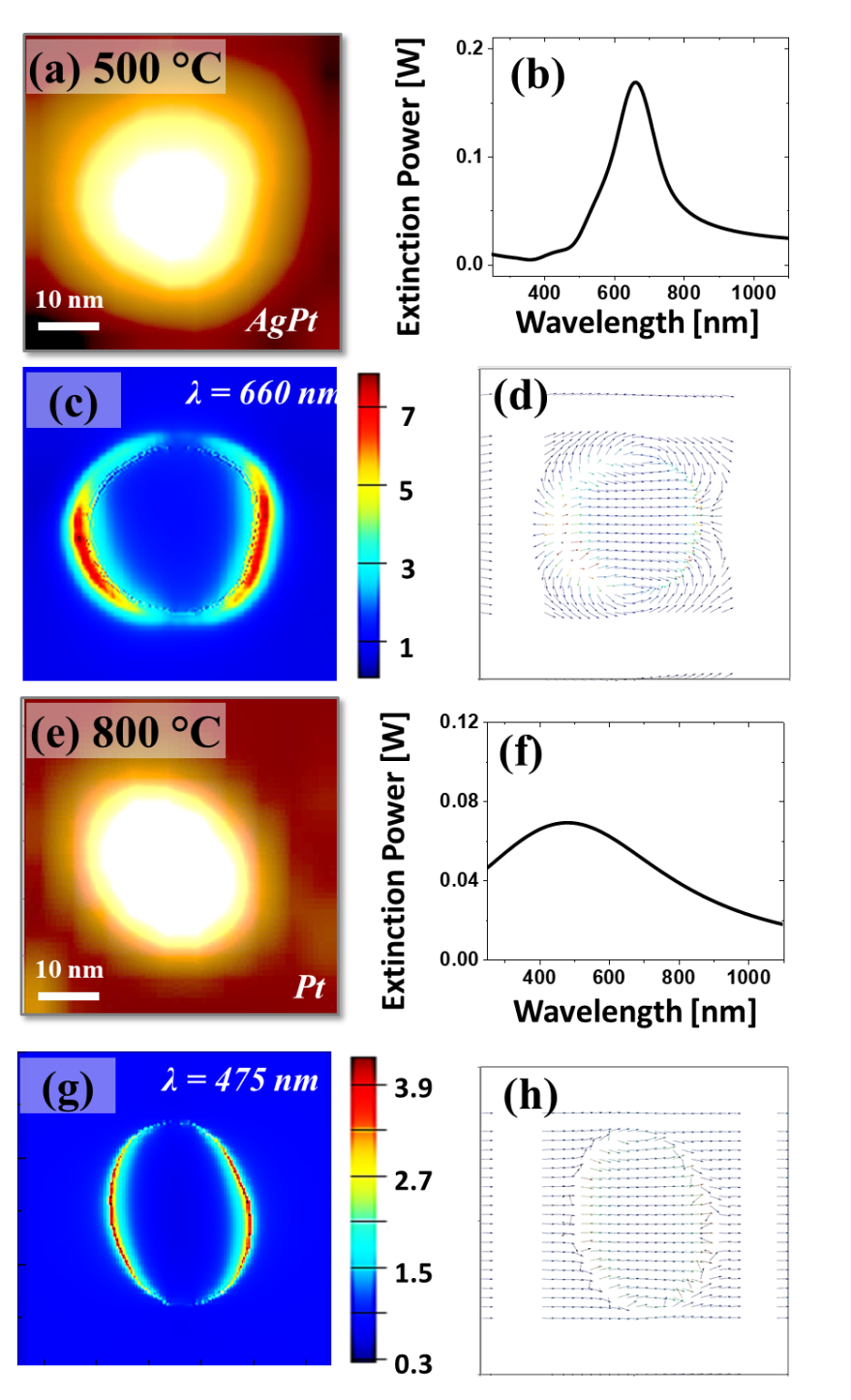
**

**Figure S4:** FDTD simulation of the typical AgPt and Pt NPs fabricated with the Ag_7nm_/Pt_1.5nm_ bilayers by annealing at 500 and 900 °C for 120 s. (a) and (e) AFM images of the typical AgPt and Pt NPs selected for the simulation. (b) and (f) Extinction power spectra of the AgPt and Pt NPs. (c) and (g) E-field profiles at resonant wavelength. (d) and (h) e-field vector plots at resonant wavelength.

**
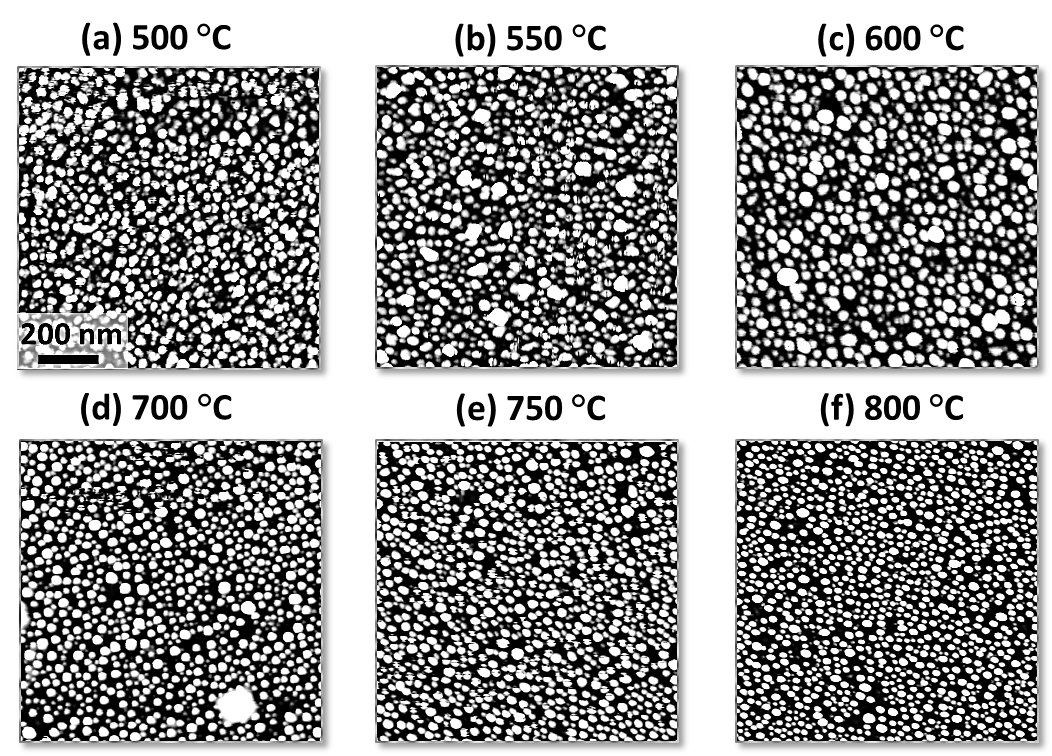
**

**Figure S5:** Evolution of AgPt and Pt NPs by the dewetting of Ag_5nm_/Pt_2.5nm_ bilayers between 500 and 800 °C for 120 s. (a) – (f) AFM top-views of 1 × 1 μm^2^.

**
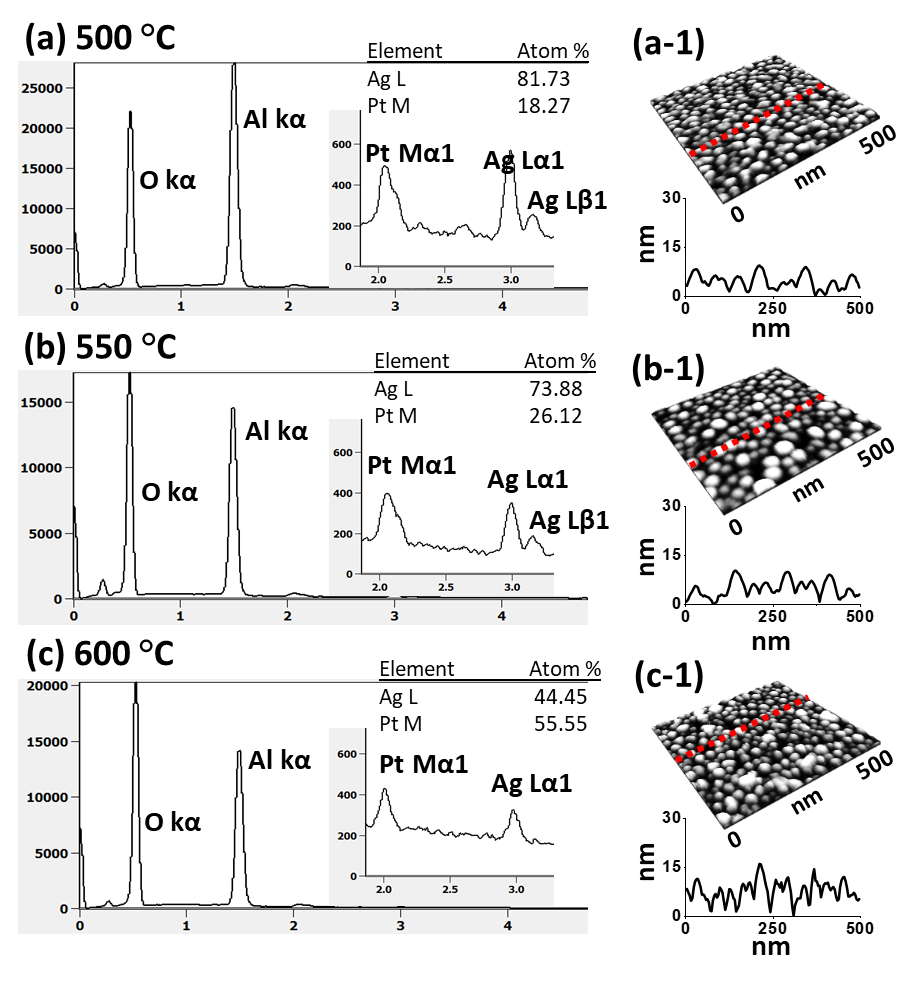
**

**Figure S6:** (a) – (d) EDS spectra of the AgPt alloy NPs fabricated with the Ag_5nm_/Pt_2.5nm_ bilayers on sapphire (0001) as a function of annealing between 500 and 600 °C for 120 s. Corresponding insets show the enlarged Pt and Ag peaks. (a-1) – (d-1) AFM side-views of 500 × 500 nm^2^ with the corresponding line profiles.

**
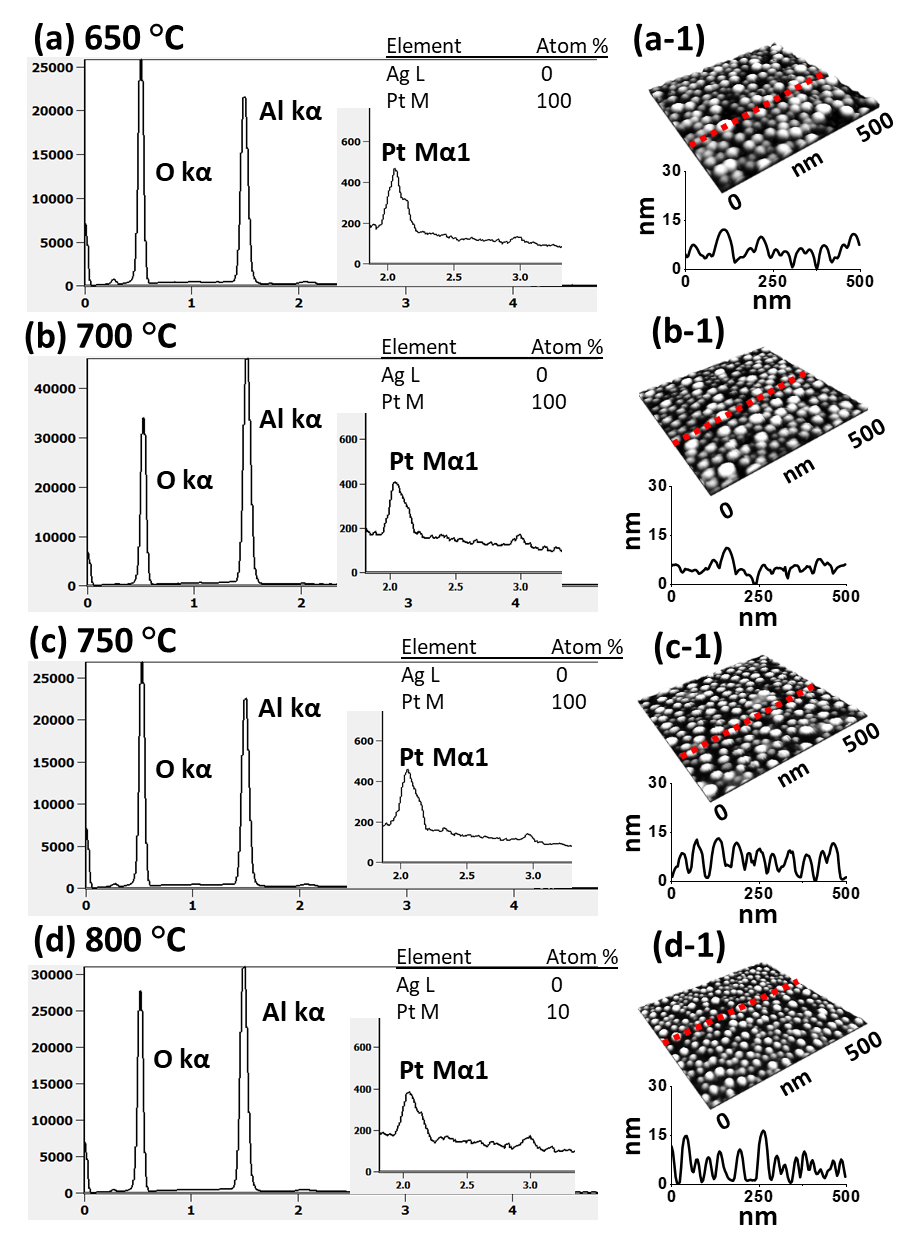
**

**Figure S7:** (a) – (c) EDS spectra of the Pt NPs fabricated on sapphire with the Ag_5nm_/Pt_2.5nm_ bilayers as a function of annealing between 650 and 800 °C for 120 s. Corresponding insets show the enlarged Pt and Ag peaks. (a-1) – (d-1) AFM side-views of 500 × 500 nm^2^ with the corresponding line profiles.


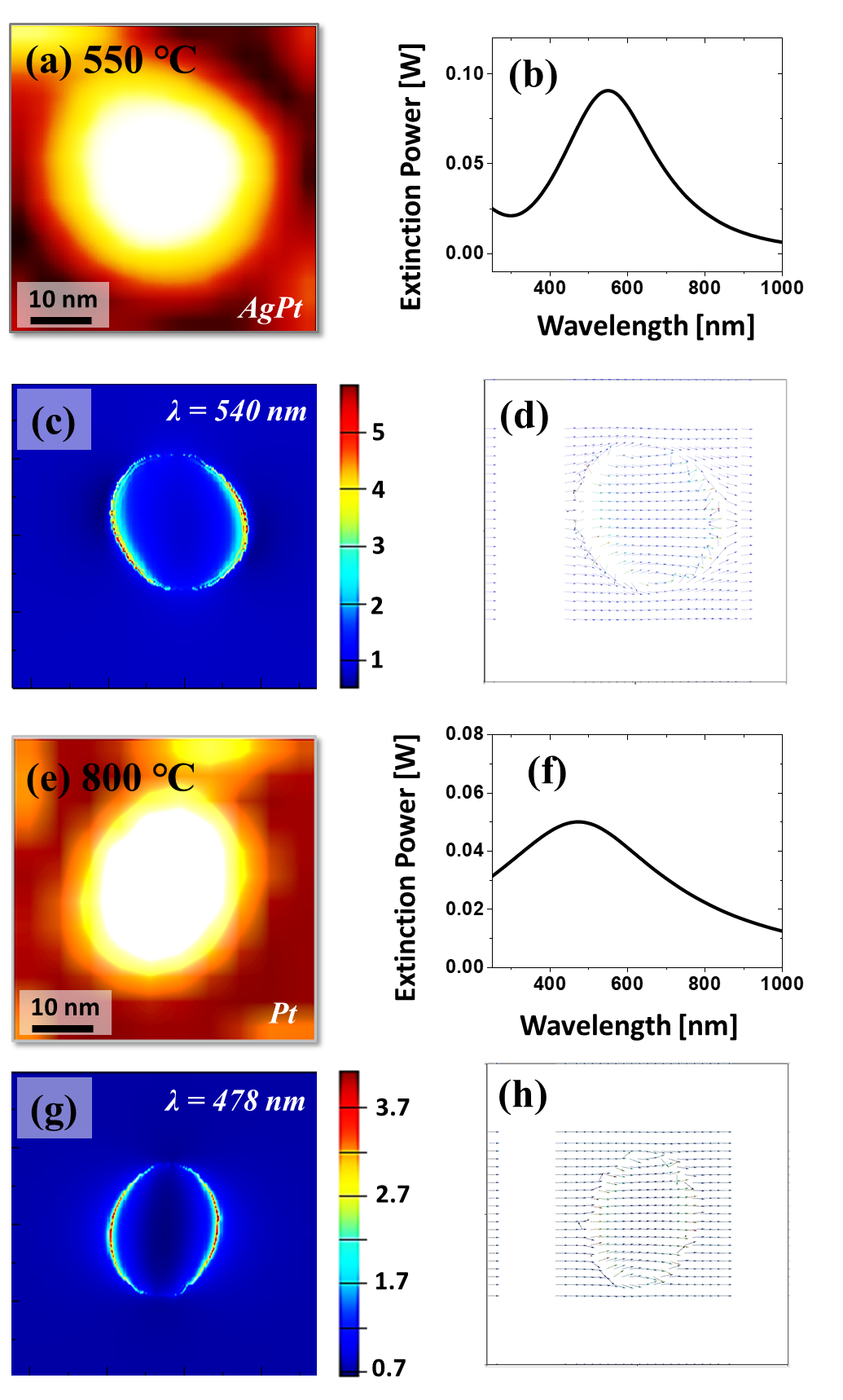


**Figure S8:** FDTD simulation of the typical AgPt and Pt NPs fabricated at 550 and 800 °C with the Ag_5nm_/Pt_2.5nm_ bilayers. (a) and (e) AFM images of the typical AgPt and Pt NPs selected for the simulation. (b) and (f) Extinction power spectra of the AgPt and Pt NPs. (c) and (g) E-field profiles at resonant wavelength. (d) and (h) e-field vector plots at resonant wavelength.


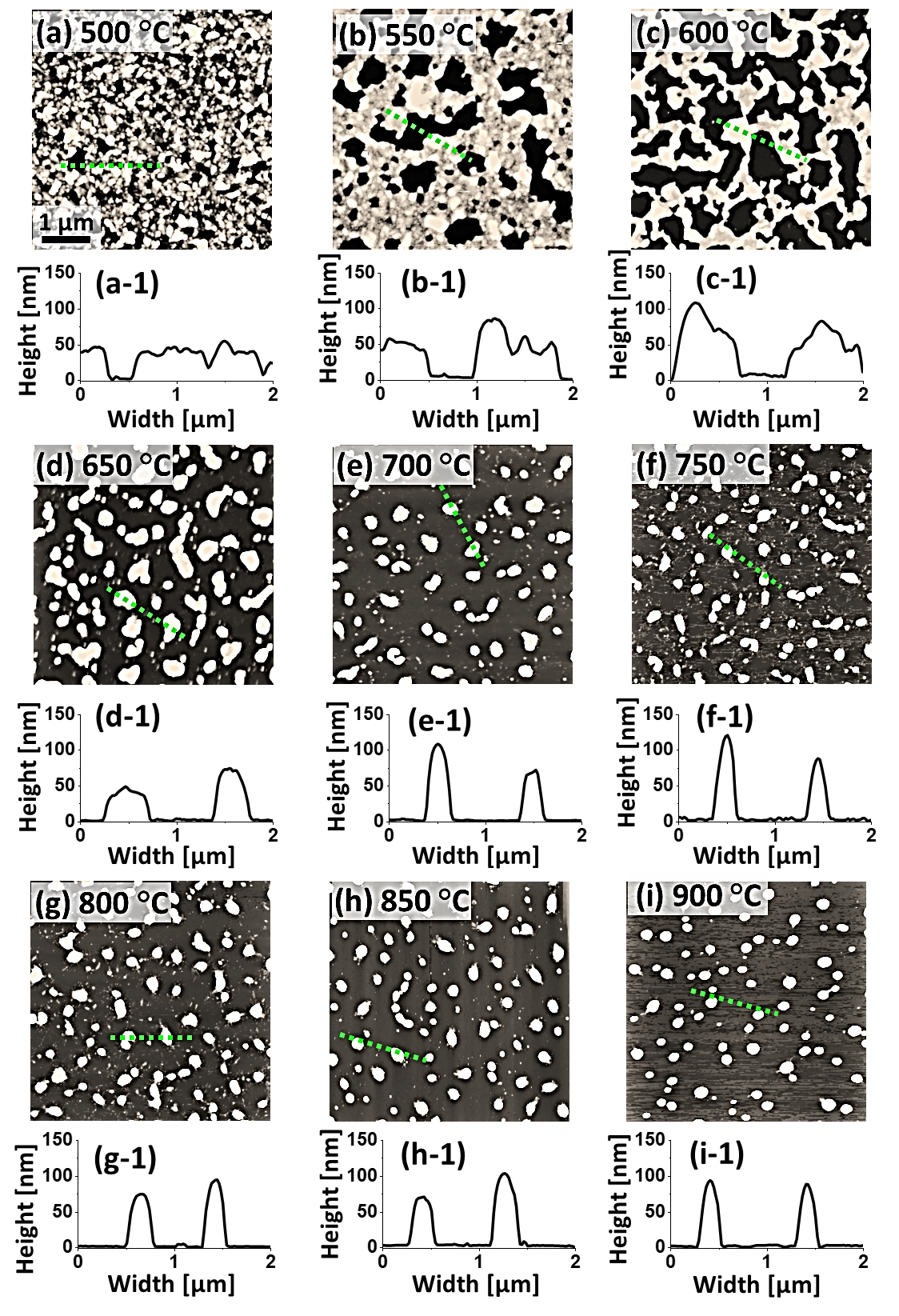


**Figure S9:** Evolution of AgPt nanoclusters and Pt NPs by the dewetting of Ag_21nm_/Pt_4.5nm_ bilayers annealed between 500 and 900 °C for 120 s. (a) – (i) AFM top-views of 5 × 5 μm^2^. (a-1) – (i-1) Cross-sectional line profiles based on the line in AFM top-views.

**
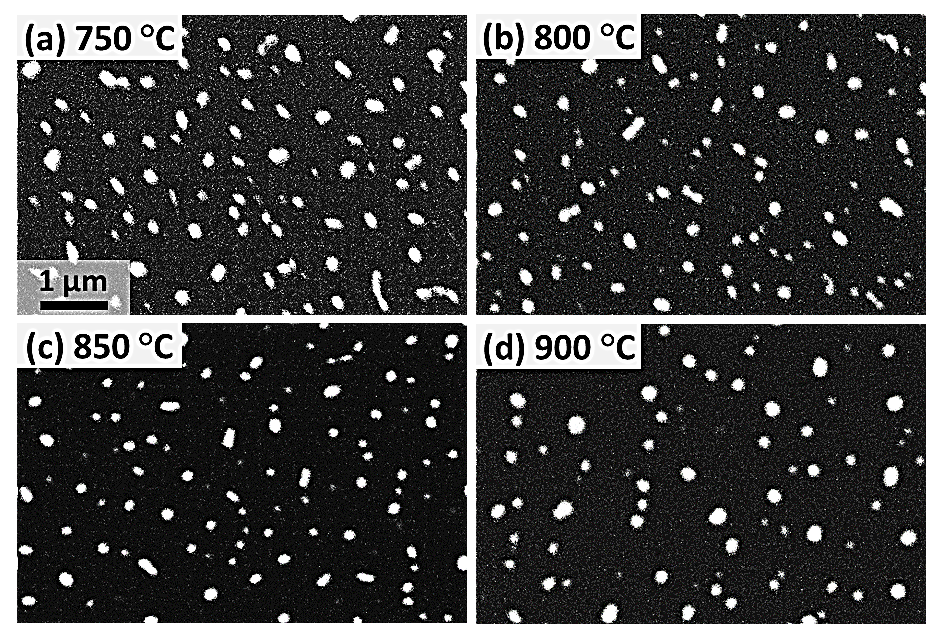
**

**Figure S10:** Scanning electron microscope (SEM) images of Pt NPs fabricated with the Ag_21nm_/Pt_4.5nm_ bilayers at different temperature as labelled.

**
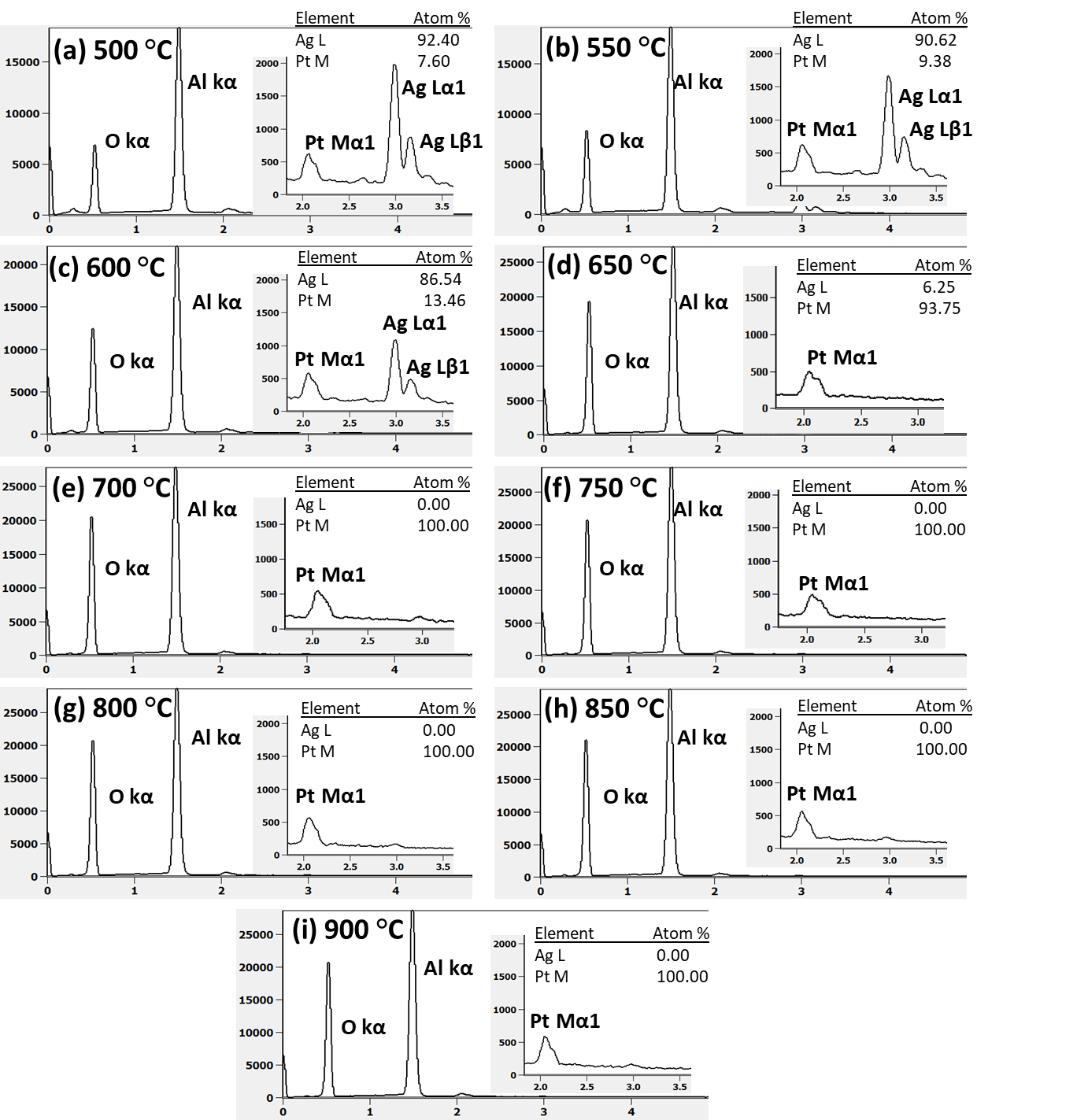
**

**Figure S11:** EDS spectra of the AgPt nanoclusters and Pt NPs nanostructures fabricated with the Ag_21nm_/Pt_4.5nm_ bilayer on sapphire (0001) by annealing between 500 and 900 °C for 120 s. Corresponding insets show the enlarged Pt Mα1 and Ag Lα1 peaks.

**
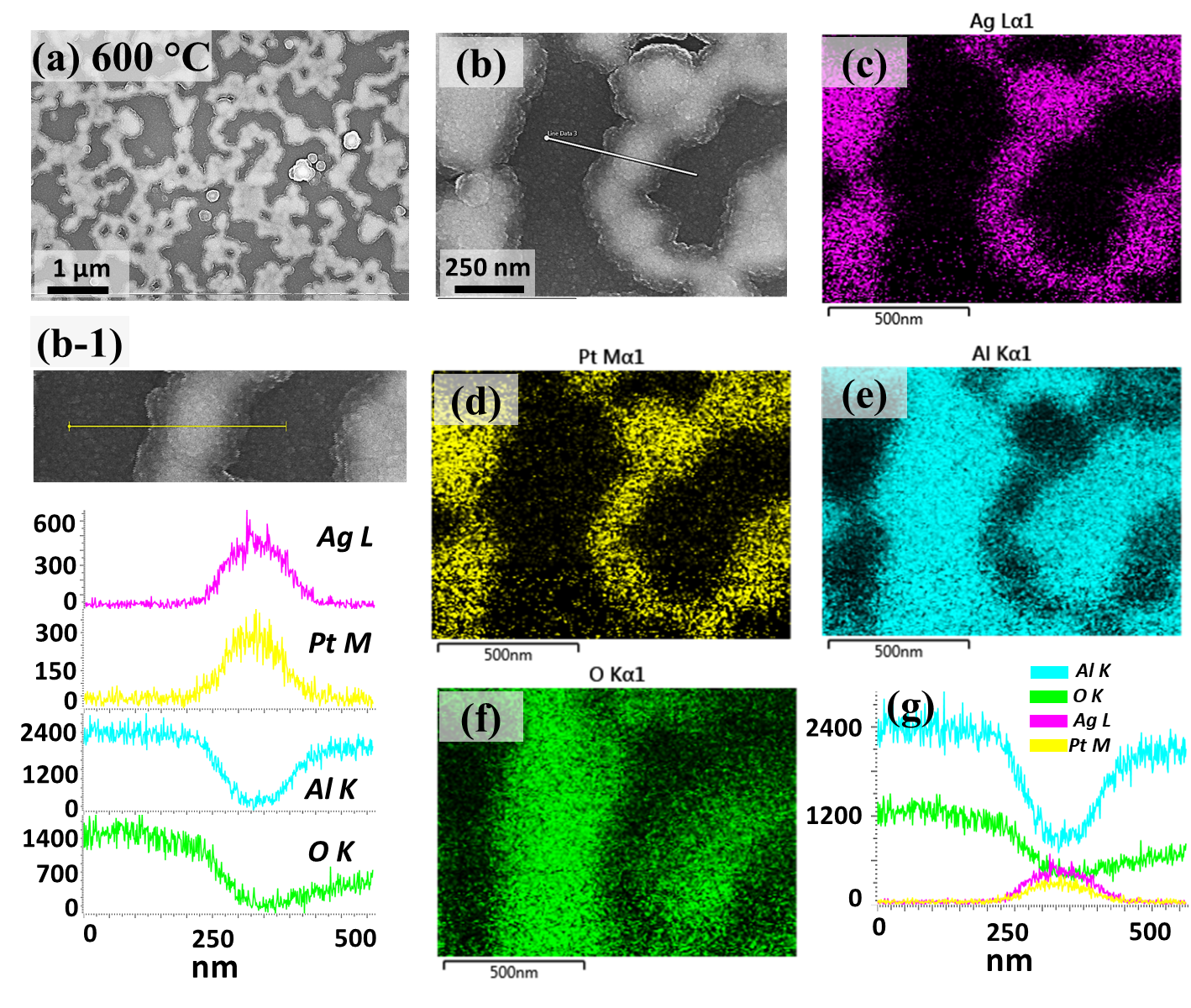
**

**Figure S12:** Detailed elemental analysis of the AgPt alloy NPs fabricated with the Ag_21nm_/Pt_4.5nm_ bilayer on sapphire (0001) by annealing at 600 °C for 120 s. (a) – (b) SEM images. (b-1) Enlarged SEM image and EDS line profiles. (c) – (f) Elemental phase maps of Ag, Pt, Al and O. (g) Combined EDS line profiles.

**
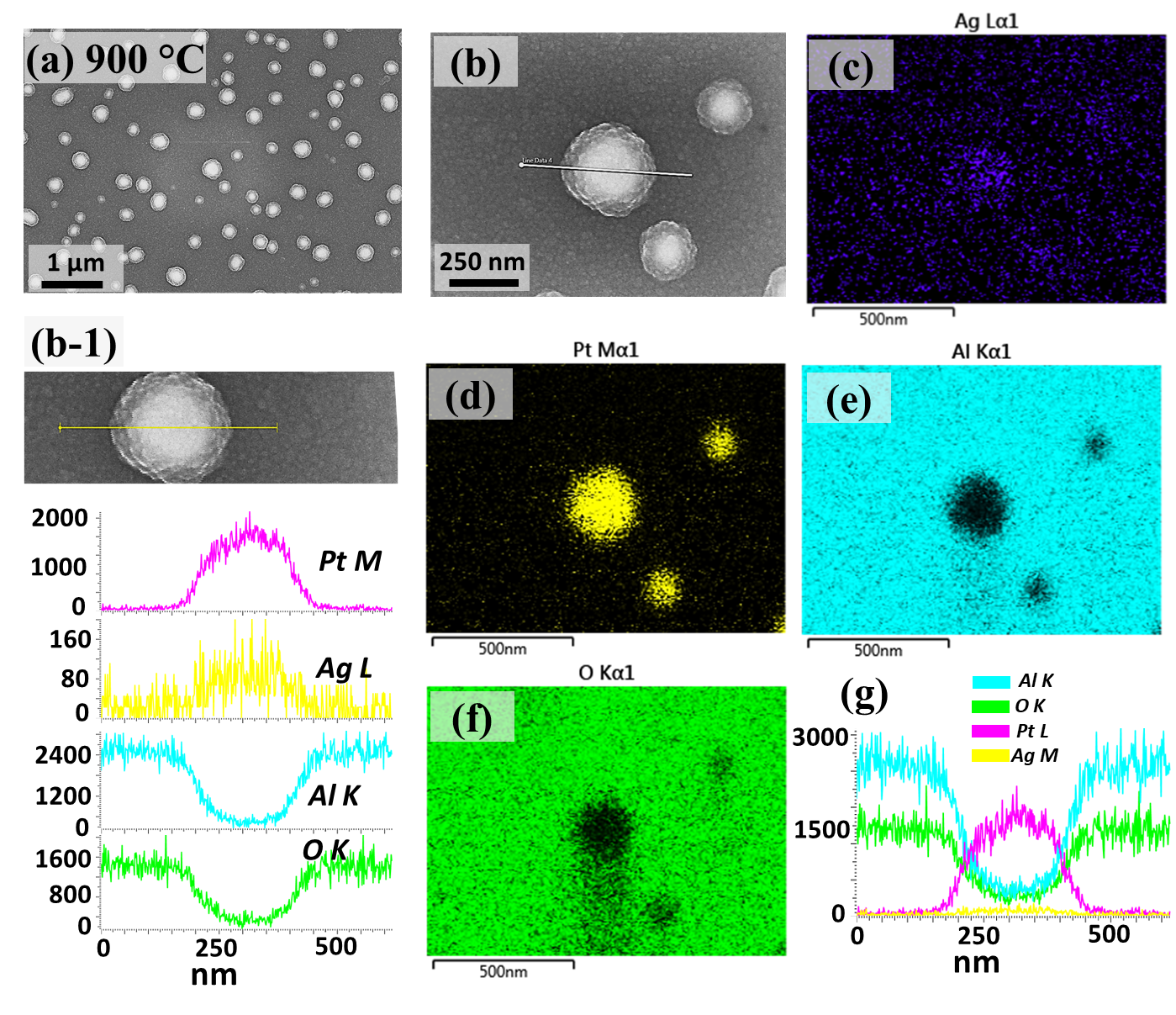
**

**Figure S13:** Detailed elemental analysis of the Pt NPs fabricated with the Ag_21nm_/Pt_4.5nm_ bilayer on sapphire (0001) by annealing at 900 °C for 120 s. (a) – (b) SEM images. (b-1) Enlarged SEM image and EDS line profiles. (c) – (f) Elemental phase maps of Ag, Pt, Al and O. (g) Combined EDS line profiles. The Ag phase map and EDS line profile are in the range of background noise, which denotes complete sublimation of Ag atoms from NPs.

**
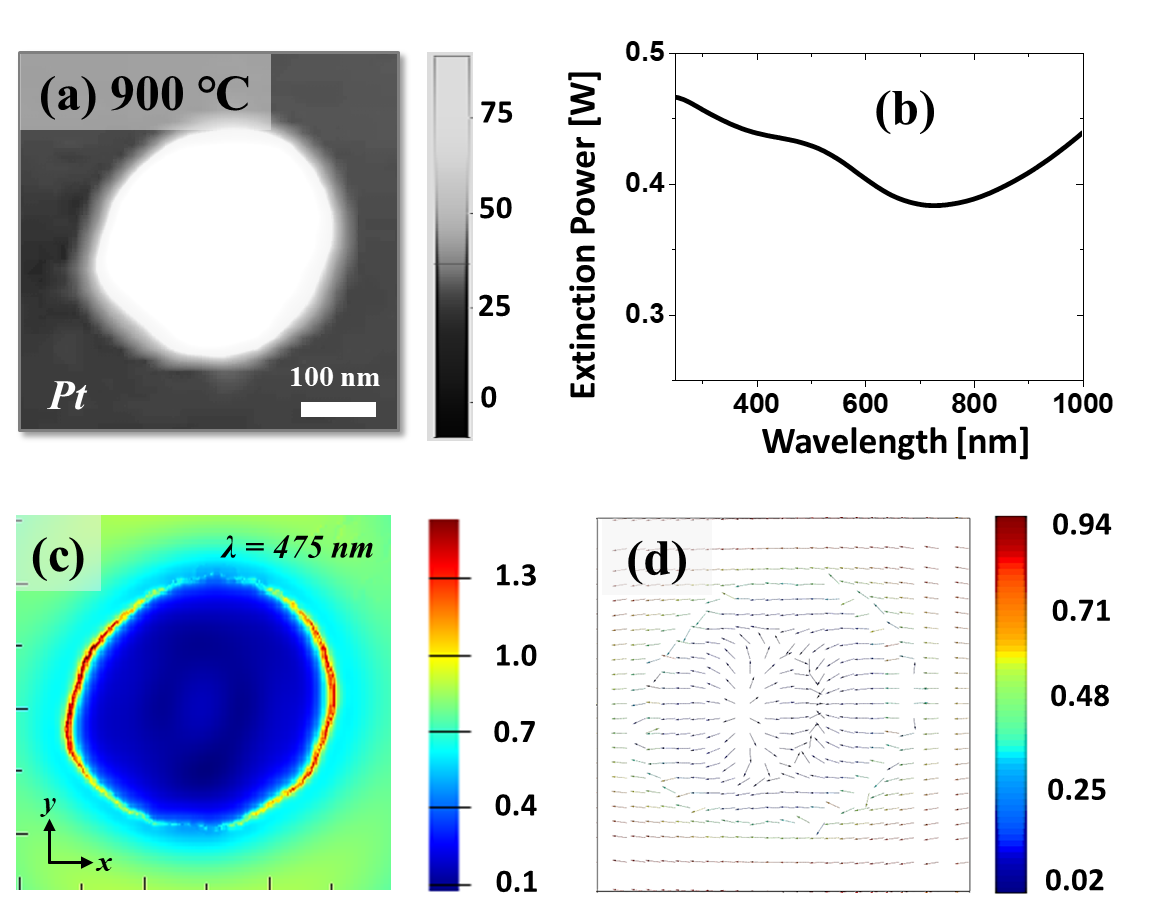
**

**Figure S14:** FDTD simulation of the typical Pt NP fabricated with the Ag_21nm_/Pt_4.5nm_ bilayer on sapphire (0001) by annealing at 900 °C for 120 s. (a) AFM image. (b) Extinction power spectra of the Pt NP. (c) E-field profiles at resonant wavelength in the VIS wavelength. (d) e-field vector plot at resonant wavelength.

**Table S1:** Summary of RMS roughness (Rq) and surface area ratio (SAR) of AgPt and Pt NPs fabricated with various Ag/Pt bilayers annealed at different temperature.

| **Temperature [°C]** | **Ag_21nm_/Pt_4.5nm_** | | **Ag_5nm_/Pt_2.5nm_** | | **Ag_7nm_/Pt_1.5nm_** | |
| --- | --- | --- | --- | --- | --- | --- |
|  | **Rq [nm]** | **SAR [%]** | **Rq [nm]** | **SAR [%]** | **Rq [nm]** | **SAR [%]** |
| **500** | 8.21 | 2.29 | 1.81 | 3.00 | 3.36 | 6.28 |
| **550** | 21.54 | 9.97 | 2.25 | 3.21 | 4.68 | 8.25 |
| **600** | 22.54 | 10.59 | 2.40 | 3.85 | 3.22 | 5.30 |
| **650** | 20.31 | 7.12 | 2.03 | 2.30 | 2.84 | 5.00 |
| **700** | 18.15 | 6.08 | 1.81 | 2.31 | 2.43 | 3.95 |
| **750** | 16.77 | 6.13 | 1.71 | 2.02 | 2.14 | 2.35 |
| **800** | 16.18 | 5.83 | 2.07 | 2.40 | 2.3 | 3.23 |
| **850** | 16.04 | 4.71 | - | - | - | - |
| **900** | 16.99 | 5.23 | - | - | - | - |

**Table S2:** Summary of average diameter (AD) and height (AH) of AgPt and Pt NPs NPs fabricated with various Ag/Pt bilayers annealed at different temperature.

| **Temperature [°C]** | **Ag_21nm_/Pt_4.5nm_** | | **Ag_5nm_/Pt_2.5nm_** | | **Ag_7nm_/Pt_1.5nm_** | |
| --- | --- | --- | --- | --- | --- | --- |
|  | **AH [nm]** | **AD [nm]** | **AH [nm]** | **AD [nm]** | **AH [nm]** | **AD [nm]** |
| **500** | - | - | 4.96 | 34.77 | 8.26 | 39.45 |
| **550** | - | - | 5.88 | 35.22 | 11.99 | 43.78 |
| **600** | - | - | 8.36 | 37.96 | 7.47 | 39.00 |
| **650** | 53.31 | 245.86 | 6.74 | 33.65 | 7.46 | 28.64 |
| **700** | 68.45 | 172.57 | 5.20 | 25.89 | 6.28 | 24.14 |
| **750** | 66.12 | 169.91 | 7.49 | 20.47 | 7.04 | 26.37 |
| **800** | 63.10 | 168.72 | 7.47 | 17.19 | 6.64 | 23.01 |
| **850** | 60.16 | 145.83 | - | - | - | - |
| **900** | 60.88 | 141.71 | - | - | - | - |
